# Systems and computational analysis of gene expression datasets reveals GRB-2 suppression as an acute immunomodulatory response against enteric infections in endemic settings

**DOI:** 10.3389/fimmu.2024.1285785

**Published:** 2024-02-16

**Authors:** Akshayata Naidu, Sajitha Lulu S.

**Affiliations:** Integrative Multi-omics Lab, Department of Biotechnology, Vellore Institute of Technology, Vellore, Tamil Nadu, India

**Keywords:** immune response, enteric infection, gene expression data analysis, network biology, machine learning methods, gene regulatory networks

## Abstract

**Introduction:**

Enteric infections are a major cause of under-5 (age) mortality in low/middle-income countries. Although vaccines against these infections have already been licensed, unwavering efforts are required to boost suboptimalefficacy and effectiveness in regions that are highly endemic to enteric pathogens. The role of baseline immunological profiles in influencing vaccine-induced immune responses is increasingly becoming clearer for several vaccines. Hence, for the development of advanced and region-specific enteric vaccines, insights into differences in immune responses to perturbations in endemic and non-endemic settings become crucial.

**Materials and methods:**

For this reason, we employed a two-tiered system and computational pipeline (i) to study the variations in differentially expressed genes (DEGs) associated with immune responses to enteric infections in endemic and non-endemic study groups, and (ii) to derive features (genes) of importance that keenly distinguish between these two groups using unsupervised machine learning algorithms on an aggregated gene expression dataset. The derived genes were further curated using topological analysis of the constructed STRING networks. The findings from these two tiers are validated using multilayer perceptron classifier and were further explored using correlation and regression analysis for the retrieval of associated gene regulatory modules.

**Results:**

Our analysis reveals aggressive suppression of GRB-2, an adaptor molecule integral for TCR signaling, as a primary immunomodulatory response against *S. typhi* infection in endemic settings. Moreover, using retrieved correlation modules and multivariant regression models, we found a positive association between regulators of activated T cells and mediators of Hedgehog signaling in the endemic population, which indicates the initiation of an effector (involving differentiation and homing) rather than an inductive response upon infection. On further exploration, we found STAT3 to be instrumental in designating T-cell functions upon early responses to enteric infections in endemic settings.

**Conclusion:**

Overall, through a systems and computational biology approach, we characterized distinct molecular players involved in immune responses to enteric infections in endemic settings in the process, contributing to the mounting evidence of endemicity being a major determiner of pathogen/vaccine-induced immune responses. The gained insights will have important implications in the design and development of region/endemicity-specific vaccines.

## 1 Introduction

Enteric infections pose major challenges to global health as diarrheal diseases remain one of the major causes of under-5 (years) mortality in Sub-Saharan Africa and South Asia (1–3). In areas of high endemicity, the suboptimal vaccine efficacy/effectiveness of oral vaccines against enteric pathogens has been quite puzzling and concerning (4–6). Several second- and third-generation enteric vaccines are under development and evaluation and can greatly benefit from the establishment of reliable correlates of protection (CoP) and/or correlates of risk (CoR) (7, 8) during the phase of clinical testing. Since the advent of high-throughput technologies, many studies have aimed at establishing gene/molecular-level signatures to induced protective immune responses against multiple vaccines (9–11) and infections instead of solely relying on antibody titers as a protective biomarker. In the course of advancements in the field quite recently, the focus has shifted towards developing and assigning gene modules (functionally associated group of genes) to vaccine-induced immunological protection against several infections (12, 13).

Particularly for enteric infections, given that endemicity plays an important role in defining vaccine-induced immune responses (14), understanding the molecular mechanisms that are underplay in endemic settings after perturbation becomes absolutely essential (15). Hence, the objective of the study was to delineate these molecular mechanisms to distinguish between immune responses in endemic and non-endemic settings (against enteric pathogens). For this purpose, we employed a robust computational and network biology pipeline for the analysis of post-infection gene expression datasets (of the host) singularly and comprehensively. Through the analysis, we expect to exhibit meaningful insight and credible molecular signatures/regulatory modules that can distinguish immune responses in these two different settings with varied pathogen prevalence. In the process, we also put forward the used pipeline as an exploratory tool for future studies that involve meta-analysis of gene expression datasets and that particularly focus on studying immune responses to pathogens.

## 2 Materials and methods

### 2.1 Data collection and conceptual framework

**Figure 1:**
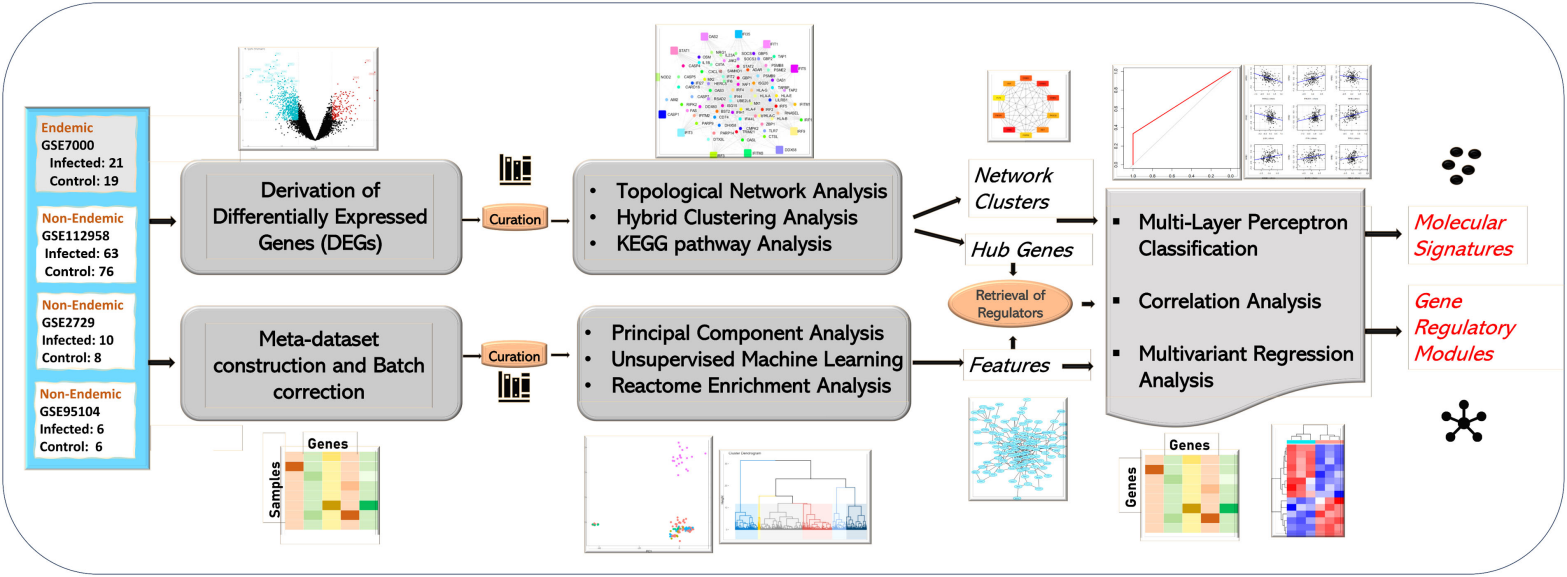
Study workflow of the analysis. The study was performed in two tiers. The first tier focused on the retrieval and topological network analysis of differentially expressed genes (at the acute stage) while the second tier focused on integration of all the infected samples in the form of a meta-dataset. The meta-dataset was used for feature selection using PCA and Random Forest Algorithm. The features/hub genes derived from the two tiers were further analyzed using correlation and multivariant regression analysis, and molecular signatures designating immune responses in endemic and non-endemic settings was derived using multilayer perceptron-based classification.

Microarray and RNASeq datasets linked to host responses to prevalent enteric pathogens—*S. typhi*, ETEC, *Vibrio cholera*, and rotavirus infections—were collected from NCBI (GEO) and EMBL-EBI (ArrayExpress) databases using the following keywords: [“Salmonella” AND “Homo Sapiens”], [“Typhoid” AND “Homo Sapiens”], [“E. coli” AND “Homo Sapiens”], and [“Rotavirus” AND “Homo Sapiens”]. A total of 125 gene expression studies were retrieved. These studies were further filtered by excluding *in vitro* studies and only clinical studies were included with infected/challenged and control groups. **Supplementary Figure S1** illustrates the detailed exclusion and inclusion criterion used for data screening and identification for the study for both endemic and non-endemic settings. The obtained gene expression datasets were segregated based on the study location and were labeled as “endemic” or “non-endemic” based on the pathogen prevalence as described in the literature. The two-tiered computational pipeline followed for the study is illustrated in **Figure 1**.

### 2.2 Data integration

For meta-dataset construction, gene expression datasets corresponding to acute stages of infection were derived from each of the studies and were integrated, and batch effect was corrected using the “sva” package’s ComBat function in R (16).

### 2.3 Differential expression analysis

Differentially expressed genes (DEGs) for each of the dataset were obtained using the “GEOquery” (17) and “limma” package (18). Briefly, gene expression datasets were retrieved for each of the studies using the “fData” function, and rows with missing values were omitted. Samples corresponding to acute responses to infections and controls were only considered for further analysis (**Supplementary Figure 1**). The four datasets were normalized using log2 transformation prior to the calculation of DEGs, which were corrected for false positives using the Benjamini & Hochberg method. The retrieved DEGs for the four tables were further filtered using logFC value (>1 and <−1) and *p*-values (0.05) and were visualized using volcano plots developed using the “ggplot2” package (19), and common and distinct DEGs were visualized using the “Venn diagram”. Missing gene symbols from these datasets were obtained using the “biomaRt” package for further analysis (20). **Supplementary File 1** provides the list of DEGs obtained for each of the cohorts in tabular format.

### 2.4 Functional enrichment analysis

The Gene Ontology database (Gene Ontology Resource) was used to prepare a master list of “biological processes” that are involved in immune responses against pathogens (**Supplementary File 2**) using the QuickGO interface (https://www.ebi.ac.uk/QuickGO/). A total of 248 biological processes were identified and used as a reference list. DEGs derived from the four datasets were individually fed to the DAVID database (https://david.ncifcrf.gov/) to derive enriched biological processes. The acquired lists (4) were manually curated to select "only" immune response-associated gene ontology terms using the drafted master list and were taken further for the analysis. Pathway enrichment analysis for all the four sets of DEGs was performed using the KEGG [KEGG PATHWAY Database (genome.jp)] (release 106.0) and Reactome (Home - Reactome Pathway Database) database (V86). Individual gene functions and associated pathways were derived from the GeneCards database (GeneCards - Human Genes | Gene Database | Gene Search).

### 2.5 Network analysis

Protein–protein interaction (PPI) networks were constructed using the STRING database [STRING: functional protein association networks (string-db.org)] and visualized and analyzed using Cytoscape (Cytoscape: An Open Source Platform for Complex Network Analysis and Visualization) plugins. The nodes of the network represent proteins and the edges represent the functional or physical associations the nodes have with each other as determined through text mining or experimental evidence and are represented and curated based on confidence scores. PPI networks were extended for up to 30 interacting partners per node (with 90% confidence score) to get a comprehensive functional understanding of the DEGs.

#### 2.5.1 Topological network analysis

Hub nodes/genes in a network can be defined as the most influential nodes in terms of connectivity and influence and were calculated using the cytohubba plugin (21). For the four constructed network, hub genes were identified using three different algorithms. While the Maximum Clique Centrality (MCC) and Density of Maximum Neighborhood Compartment (DMNC) algorithms revealed nodes with maximum connectivity that were relevant in understanding influential proteins for each of the networks, the Bottleneck algorithm was especially important in extracting nodes that connected different subnetworks. The employed algorithms are detailed as follows:

- MCC is a local-based method for topological analysis where the MCC score for a node or *MCC*(*v*) is defined as *MCC*(*v*)*=∑C*∈*S*(*v*)(*|C|*−1)!, where *S*(*v*) is the collection of maximal cliques that contain *v*, and (|*C*|−1)! is the product of all positive integers less than |*C*|.
- DMNC is also a local-based method for topological analysis where the DMNC score or *DMNC*(*v*) of a particular node is defined as *DMNC*(*v*) *= |E*(*MC*(*v*))*|*/*|V*(*MC*(*v*))*|^ε^*, where *ε* = 1.7, *MC*(*v*) is a maximum connected component of the *G*[*N*(*v*)], and *G*[*N*(*v*)] is the induced subgraph of *G* by *N*(*v*) (total set of nodes). *V* is a collection of nodes and *E* is a collection of edges.
- The Bottleneck algorithm, on the other hand, is a global-based method for topological analysis where the Bottleneck score *BN*(*v*) is defined as *BN*(*v*)*=∑s*∈*Vps*(*v*), where *ps* (*v*) = 1 if more than |*V*(*Ts*)|/4 paths from node *s* to other nodes in *Ts* meet at the vertex *v*; otherwise, *ps*(*v*) = 0.

The PPI network clusters were detected using the MCODE algorithm available in the ClusterViz plugin in Cytoscape (22). The algorithm maps highly interconnected subnetworks of a network. In this algorithm, seed vertices are expanded based on the local neighborhood density and the density of the prospective cluster.

### 2.6 Feature selection through unsupervised machine learning algorithm

Firstly, principal component analysis (PCA) was performed on the constructed meta-dataset (section 2.2) to characterize the variance of gene expression profile in endemic and non-endemic settings. PCA is a dimension reduction technique used to derive key insights into big datasets based on the covariance of the variables involved based on the derived eigenvectors and values. Mathematically, covariance between two variables is defined as:


$$\text{Cor}(x,y)\;=\text{ Sum ((}x_{i}–x*\text{) (}y_{i}–y*\text{))}/N$$

where *x* and *y* represent two variables, x* and y* represent their respective means, and *N* represents the total sample size of the study. PCA is generally used as a preliminary step to observe the underlining patterns of the large datasets and how these patterns are correlated with the phenotype/outcomes under consideration. The analysis was performed using the “prcomp” function in R.

Secondly, feature selection was performed using the Random Forest algorithm-based wrapper method that distinguished between gene expression profiles (with common 6,543 genes) from endemic and non-endemic settings using the “Boruta” package (23). Random Forest belongs to the family of decision trees where, based on numerical estimates, independent decision trees are constructed and evaluated for optimal classification performance. The importance of a variable is calculated based on the loss in accuracy in classification when the variable is dropped in a series of random permutations. The importance of each variable is determined using the *Z* score in the Boruta package. Mathematically, the *Z* score in the Boruta package can be defined as the average of the difference in real and predicted values of a variable (or the loss of accuracy) divided by the standard deviation. The higher the loss of accuracy computed for a variable, the poorer it seemed to have performed, and *vice versa*. The parameters used in the algorithms are optimized based on trial and error and are hence auto-optimized or auto-tuned.

Thirdly, hybrid clustering (using components of both k-means and hierarchical clustering algorithms) was performed on the logFC values of common genes between the four cohorts using the “FactoMineR” package (24). In hybrid clustering, small clusters are initially formed using the k-means algorithm (centroid-based clustering), which are later clustered on a larger scale based on the maximal distance between the formed clusters and come under hierarchical or connectivity-based clustering. Mathematically, k-means clustering relies on the calculation of Euclidean distance between two variables in order to assign variables to specific centroids. The Euclidean distance between two variables is computed as:


$$d^{2}(x,y\text{) }=\text{ (}x_{1}–y_{1}{)}^{2}+\text{ (}x_{2}–y_{2}{)}^{2}+\text{ (}x_{n}-y_{n}{)}^{2}$$

where *x* and *y* represent the two variables (their values) in a plane and *n* represents the number of samples. On the other hand, maximal distance between two clusters in hierarchical clustering is computed as:


$$d(p,q)\;=T_{pq}/N_{p}+N_{q}$$

where *p* and *q* represent the two clusters, *T* represents the sum of the pairwise distances between the two clusters, and *N* represents the number of variables in the respective clusters.

The features/attributes/genes derived from the two algorithms (clustering and Random Forest) were used for the construction of the PPI network using the STRING database, and hub genes were retrieved through topological network analysis performed using the cytohubba plugin (**Figure 1**).

### 2.7 Machine learning based classification

Hub genes derived through the methods described in sections 2.5 and 2.6 specifically were used for the construction of classification models using the meta-dataset to distinguish between the endemic and non-endemic (infected) groups using the multilayer perceptron (MLP) algorithm on the WEKA platform with threefold cross-validation. Neural networks, specifically MLP, are well documented in the literature as good classifiers when gene expression datasets are used as input (25, 26). MLP is a deep machine learning algorithm that consists of an input layer, an output layer, and a hidden layer, and the neural network is trained using a feed-forward pathway. The activation function used for training was a sigmoid logistic function represented as:


$$F(x)\;=\;1/(1\;+\;{\text{e}}^{-\text{x}})$$

which is a nonlinear function and represents an input variable in the range of 0 to 1. Activation functions are used to gauge and legitimize specific neurons or nodes of the neural network during training based on the weight and bias they hold for the classification. Thereafter, confusion matrices representing the performance of the classification were computed and visualized. The confusion matrix summarizes true positive (TP), false positive (FP), false negative (FN), and true negative (TN) values predicted by the model. The confusion matrix is used to compute Accuracy and Recall of the built classifier, where


Accuracy = TP + TN/(TP + TN + FP + FN)

Recall = TP/TP + FN

Accuracy represents the instances (out of total) where the classification predictions were correct, while Recall represents instances where the predictions were correct as compared to total positives (TP + FN). Genes were ranked based on the accuracy score of their respective models.

### 2.8 Correlation analysis

Correlation modules were retrieved using the “azolling/EBmodules” package (https://github.com/azolling/EBmodules) from the constructed meta-dataset, and modules with high-performing genes from the section above were identified (27). The algorithm behind the package combines gene–gene correlation matrices derived from different sets of microarray datasets with the sample-gene architecture using the Fischer transformation. From this constructed common correlation matrix, highly correlated genes or modules are derived using hierarchical clustering algorithm. The optimal number of modules to be derived from the correlation matrix is decided using the Gap statistical method that is discussed in detail elsewhere (https://joey711.github.io/phyloseq/gap-statistic.html), and for each cluster, Gap(*k*) is computed using:


Gap(k) =(1/B) sum(log(W*) – log (Wk)

### 2.9 Multiple regression analysis

Genes correlated to high-performing genes (based on MLP classification) (or part of shared network clusters from section 2.5) and retrieved transcriptional factors for each of these genes were used for the construction of multivariant regression (MVR) models in R. MVR involves the prediction of a dependent variable based on a set of independent variables (instead of a single variable that is used in the single-variant regression analysis). Mathematically, regression models can be defined as:


$$Y=\beta 0+\beta 1x_{i}+\epsilon_{i}$$

where *Y* represents the dependent variable under investigation and *x* represents independent variables, while *β*0 and *β*1 represent the intercept and parameter of the model, respectively, and ϵ represents standard error. “*i*” indicates the number of independent variables being tested for the prediction of *Y*. For the highly influential genes derived from the steps above, MVR models were retrieved using a combinatorial approach where genes found to be correlated or associated with these genes of interest (throughout the analysis) were treated as independent variables to derive the best-performing model that could predict the pattern of expression of these influential genes. The aim of the analysis was to gain a deeper understanding of the underlying molecular mechanisms for the construction of robust gene regulatory modules associated with identified molecular signatures. MVR has been recently suggested as a robust method for deriving gene regulatory networks from gene expression datasets (28). The analysis was performed using the “lm” function in R.

### 2.10 Regulatory network inference

MVR models constructed in the above step with *R* ^2^ value > 0.50 were used for the inference of gene regulatory modules.

## 3 Results

Based on the criteria discussed in section 2.1, four gene expression studies—GSE7000, GSE112959, GSE2729, and GSE95104—were selected for the analysis. Here, GSE7000 study datasets were retrieved from subjects in Vietnam (a country endemic to *S. typhi* infection), whereas the latter three were from non-endemic settings. GSE112958 study datasets were derived from *S. typhi-*challenged adults in a controlled study conducted in Oxford (UK). GSE2729 datasets were retrieved from rotavirus-infected children from the USA and GSE95104 datasets were derived from ETEC-infected subjects from the USA (**Table 1**). Datasets from the earliest time points (post-symptom onset) for each of the four studies were used for the retrieval of DEGs and for the construction of the meta-dataset (**Supplementary Figure 1**). An integrated dataset (meta-dataset) with 6,543 common genes was constructed, and the batch effect was corrected for a total of 208 samples (all infected samples from the four datasets) (**Supplementary Files 5** and **6**) for meta-analysis of gene expression datasets. An online accessible processed dataset with 20 samples from GSE69529 (RNASeq) was reserved for validation (**Supplementary File 7** and **Supplementary Figure S1**).

**Table 1:** GEO Accession ID with description of the four microarray datasets used in the study along with a rnaseq dataset used for validation.

GEO Accession ID	Microarray platforms	Pathogen	No. of samples	Study population	Location	Reference
**GSE2729**	Affymetrix Human Genome U95 Version 2 Array	Rotavirus	23	Children, infected	USA	(29)
**GSE95104**	Affymetrix Human Genome U133A 2.0 Array	ETEC	72	Adults, challenged with unattenuated ETEC strain	USA	(30)
**GSE7000**	Stanford Human cDNA Microarray	*S. typhi*	183	Adults, INFECTED	Vietnam	(31)
**(GLP4858)**						
**GSE112958**	Illumina HumanHT-12 V4.0 expression bead chip	*S. typhi*	178	Adults, challenged with *S. typhi* Quailes strain	UK	(*Diagnostic Host Gene Signature for Distinguishing Enteric Fever from Other Febrile Diseases—EMBO Molecular Medicine*, 2019)
**GSE69529**	Illumina HiSeq 2500	Multiple	204	Children, infected with multiple pathogens	Mexico	(32)

*RNA was extracted from PBMC samples in the first three studies and from the whole blood samples in the fourth study.

### 3.1 Retrieved differentially expressed genes, enriched pathways, and modules

**Figure 2:**
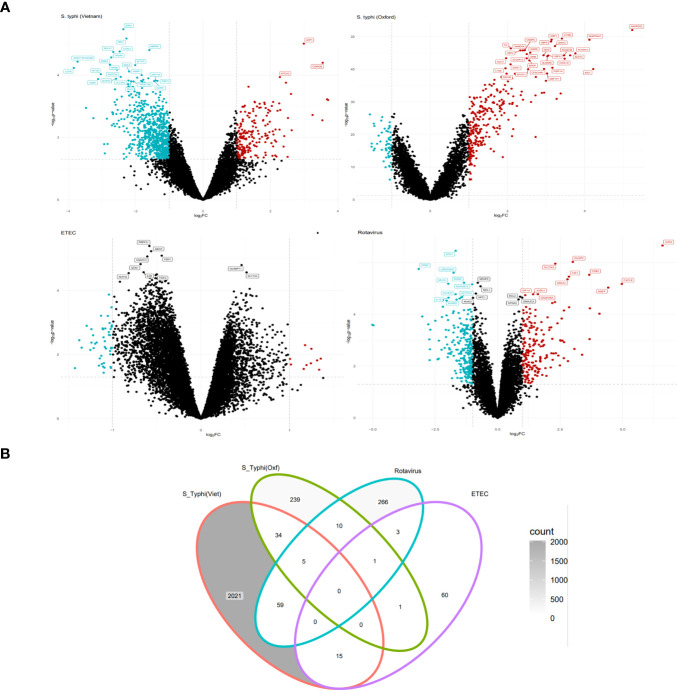
EXTRACTION OF DIFFERENTIALLY expressed genes (DEGs) (Tier 1). **(A)** Volcano plots depicting upregulated and downregulated genes derived from GSE7000, GSE112958, GSE95104, and GSE2729 (clockwise). **(B)** Venn diagram representing common and specific genes between the cohorts.

At the early stage of infection, in the *S. typhi* cohort, there were 887 upregulated genes while there were 1,249 downregulated genes. For the *S. typhi* (Oxford) cohort, there were 258 upregulated genes and 34 downregulated genes. For the Rotavirus cohort, there were 139 upregulated genes and 207 downregulated genes. For the ETEC cohort, there were 80 upregulated genes and no genes were downregulated based on the set criterion (**Supplementary Table S1**). The retrieved DEGs from the four cohorts were illustrated as Volcano plots (**Figure 2A**). Briefly, for the *S. typhi* (Vietnam) cohort, there was upregulation of markers of activated lymphocytes and mediators of the NOTCH signaling pathways, and downregulation of mediators involved in acute inflammatory responses. For the *S. typhi* (Oxford) cohort, highly upregulated genes were inferred to an interferon-mediated inflammatory response along with the mediation of T-cell chemotaxis. For the Rotavirus cohort, we found upregulation of inflammatory cytokines, and for the ETEC cohort, we found upregulation of mediators involved in early stages of inflammation. In terms of numbers, we found the least number of DEGs in the ETEC cohort and the highest number of DEGs in the *S. typhi* (Vietnam cohort). While the *S. typhi* (Vietnam) cohort had 59 DEGs in common with the Rotavirus cohort, there were only 34 DEGs common with the *S. typhi* (Oxford) cohort (**Figure 2B**).

Functional enrichment analysis was performed to gain biological insight into acute responses to pathogen in endemic and non-endemic cohorts. Kyoto Encyclopedia of Genes and Genomes (KEGG) pathway analysis on the DEGs acquired for the *S. typhi* (Vietnam) cohort revealed significant enrichment of multiple intracellular signaling pathways, top among which were the cGMP-PKG signaling pathway and the Calcium signaling pathway (**Supplementary Table S2**). Interestingly, pathway enriched analysis of “both” up-and downregulated genes separately for this cohort revealed enrichment of T-cell receptor signaling (at the acute state of infection). While CD40L, PI3K, SOS, HRAS, and PLC genes were upregulated, LCK and GRB2 were downregulated (**Supplementary Figure S2**) along with the downregulation of major signaling pathways conventionally associated with acute inflammatory responses (toll-like receptor signaling and cytokine/chemokine signaling pathway) (**Supplementary Figure S3**). For the *S. typhi* (Oxford) cohort, sensory signaling pathways—NOD-like receptor signaling pathways and the Cytosolic DNA-sensing signaling pathway—along with intracellular pathways involved in antigen processing and presentation were significantly enriched. On the other hand, in the Rotavirus cohort, enrichment of major inflammatory signaling pathways was observed upon KEGG pathway enrichment analysis. Importantly, pathways associated with PRR signaling and TCR/BCR signaling were also significantly enriched for this cohort. For the ETEC cohort, given the low number of DEGs derived for this cohort, no enriched KEGG signaling pathways were detected (**Supplementary Table S2**).

**Figure 3:**
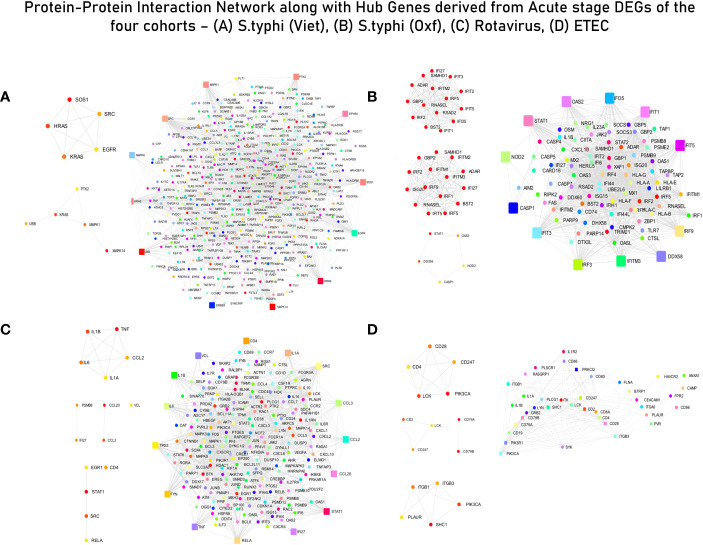
Protein–protein interaction (PPI) network with interacting partners (IPs) (Tier 1). **(A)** *S. typhi* (Vietnam) cohort—MCC hub genes and Bottleneck hub genes. **(B)** *S. typhi* (Oxford) cohort—MCC hub genes, DMNC hub genes, and Bottleneck hub genes. **(C)** Rotavirus cohort—MCC hub genes, DMNC hub genes, and Bottleneck hub genes. **(D)** ETEC cohort—MCC hub genes, DMNC hub genes, and Bottleneck hub genes (confidence score: 0.90).

Enrichment and curation of GO biological processes based on the master list (section 2.4) yielded a total of 91 immune response-associated modules for the *S. typhi* (Vietnam) cohort, 117 modules for the *S. typhi* (Oxford) cohort, 118 modules for the Rotavirus cohort, and 6 modules for the ETEC cohort. The top curated enriched terms for the *S. typhi* (Vietnam) cohort were “inflammatory response”, “positive regulation of cell migration”, “cell surface receptor signaling pathways”, “response to xenobiotic stimulus”, and “neutrophil chemotaxis”. Curated terms for *S. typhi* (Oxford) were “defense response to virus”, “innate immune response”, “response to virus”, “negative regulation of viral genome replication”, and “positive regulation of interferon beta production”. For the Rotavirus cohort, the top enriched biological processes (after curation) were “chemokine-mediated signaling pathway”, “cellular response to lipopolysaccharide”, “negative regulation of MAPK cascade”, “cytokine mediated signaling pathway”, and “negative regulation of type 2 immune response”. For the ETEC cohort, the top enriched (curated) terms were “regulation of phosphatidylinositol 3-kinase signaling”, “positive regulation of innate immune response”, “immune response”, “acute-phase response”, “regulation of immune system process”, and “T-cell activation”. Genes associated with curated GO terms were taken ahead for PPI network construction and analysis (**Figure 3**).

Overall, through the KEGG enrichment analysis, we found peculiar dysregulation of the TCR receptor signaling pathway in the endemic cohort as compared to the non-endemic cohort (**Supplementary Figures S2** and **S3**). Furthermore, although all the four cohorts showed enrichment of biological processes involved in host responses to the pathogen and acute inflammatory responses, we observed specific enrichment of modules associated with cell migration in the endemic cohort.

### 3.2 Hub genes and network clusters

The list of genes derived for each of the cohorts after module screening and identification (**Supplementary File 3**) was used as input for the construction of PPI networks (as described in section 3.1) to retrieve genes of high influence or connectivity (hub genes) in immunologically relevant gene ontologies (for the four cohorts). Although PPI networks were constructed using a curated set of genes with high immunological relevance, for the *S. typhi* (Vietnam) cohort, topological analysis of the network did not derive any hub genes conventionally associated with immune responses. In fact, majority of the hub genes derived from the three topological algorithms were associated with cell cycle signaling (SOS1, HRAS, and KRAS), EGFR receptor-associated (EGFR and SRC), and MAPK/Erk (MAPK6/14) signaling pathways (**Supplementary Table S3** and **Figure 3A**). For immune responses in the *S. typhi* (Oxford) cohort, hub genes using the MCC and DMNC algorithm were IRF1, IFIT1/3/4, and IFI35, and IRF1/4, IFIT5, and IFITM1/3, respectively. Both of these sets of genes are essential components of interferon-mediated signaling pathways (**Supplementary Table S3** and **Figure 3B**). For the Rotavirus cohort, major inflammatory mediators—RELA, JUN, STAT3, CREBBP, IL6R, CXCL3/8, TNF, and STAT1—were revealed as hub genes of the constructed network (**Supplementary Table S3** and **Figure 3C**). In the ETEC cohort, degree-based topological algorithms (MCC and DMNC) revealed adaptors and receptors involved in TCR (CD28, CD2, CD28, and CD247) and BCR (CD79A/B) signaling pathways as essential hub genes in the elicited immune response (**Supplementary Table S3** and **Figure 3D**).

Network clusters derived from the four pathogen-specific PPI networks were filtered based on their clustering scores (>5 score); three clusters were retrieved from the *S. typhi* (Vietnam) cohort and one cluster (with a score of 40.55) was retrieved from the *S. typhi* (Oxford) cohort. From the Rotavirus cohort, three clusters were retrieved and two clusters were retrieved from the ETEC cohort. Fully annotated clusters are illustrated and described in **Supplementary Figure S2** and **Supplementary Table S4**, respectively. Briefly, the highest-performing network cluster from the Vietnam cohort was enriched with genes belonging to the growth receptor signaling pathway (EGF, EGFR, MAPK, RHOA, KRAS, HRAS, GRB2, SHC, and PTPN11) and T-cell receptor signaling pathway (GRB2, LCK, SRC, MAPK, and HRAS). The highest-performing cluster in the *S. typhi* (Oxford) cohort was enriched with genes belonging to interferon-induced mediators, that in the Rotavirus cohort was enriched with cytokines and chemokines, and that with the ETEC cohort was enriched in surface mediators of lymphocyte signaling. Considering that the functional enrichment analysis pointed towards a dysregulated TCR signaling specifically in the *S. typhi* cohort, the highest performing cluster from the *S. typhi* (Vietnam) cohort (which was enriched with genes from tcr and growth factor receptor signalling) was considered as a distinguishing and peculiar feature of acute immune responses in the endemic cohort.

### 3.3 Features distinguishing immune responses in endemic and non-endemic settings

**Figure 4:**
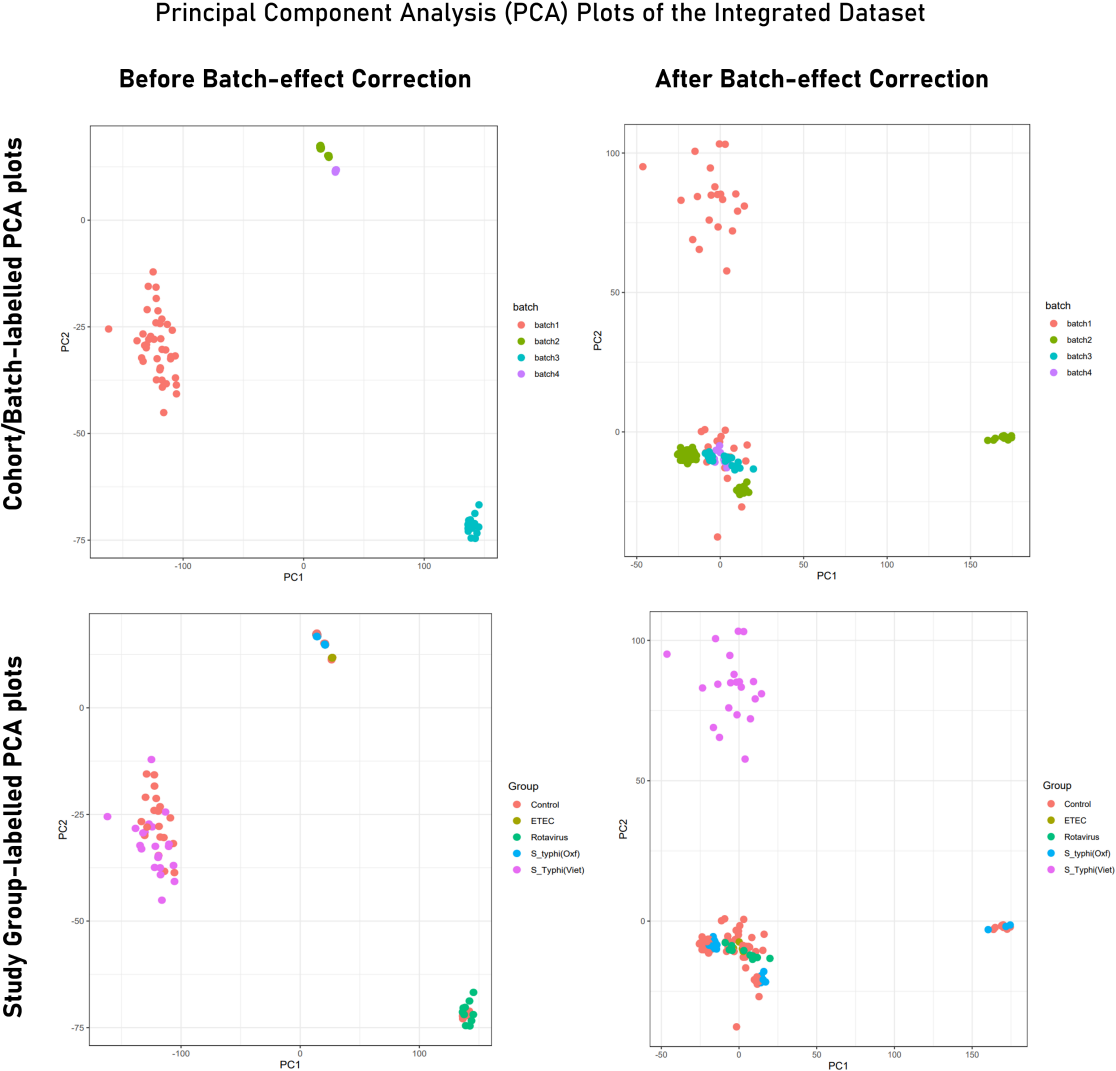
PCA plot illustrating variance in gene expression profiles (Tier 2) before and after batch correction. While PCA plots in the upper panel are labeled to indicate samples from different experiments/cohorts/batches, PCA plots from the lower panel are labeled with different study groups (infected and control). Here, Batch 1 = *S. typhi* (Viet) cohort (endemic); batch 2 = Rotavirus cohort; batch 3 = *S. typhi* (Oxf) cohort; batch 4 = ETEC cohort.

**Figure 5:**
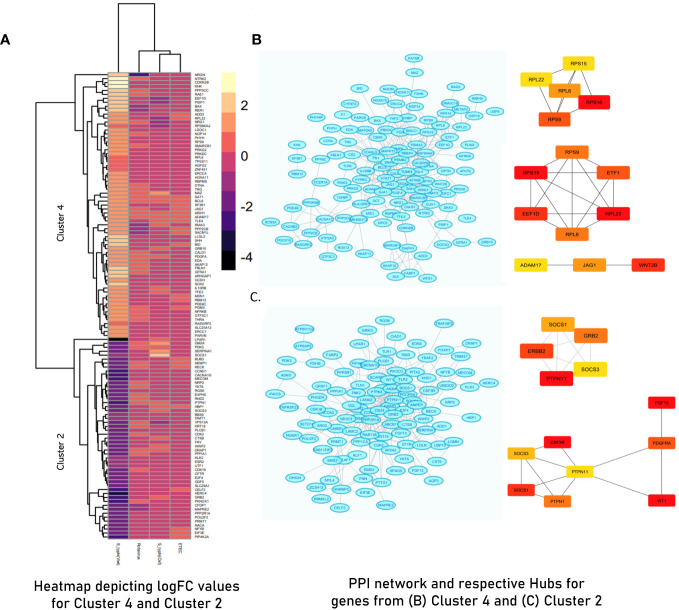
**(A)** Heatmap illustrating Cluster 2 and Cluster 4 derived from hybrid clustering (Tier 1) where yellow depicts logFC >2 and violet depicts logFC< −4. **(B)** STRING network and derived hub genes for Cluster 4. **(C)** STRING network and derived hub genes for Cluster 2 where light blue color nodes depict members of Cluster 4 and Cluster 2, respectively. For both clusters, hub genes were identified using MCC (up) and DMNC (down) algorithms where red-orange-yellow-colored nodes depict hub genes with high scores as calculated by respective algorithms with red- colored nodes depicting the highest scoring genes.

PCA of the integrated gene expression dataset (meta-dataset) revealed a high degree of variance in PC1 and PC2 and was performed to gauge covariances/eigenvectors corresponding to the four cohorts. While variance in component 1 was attributable to the differences in the gene expression profile between an adult cohort and a child cohort, variance in component 2 can be attributed to gene expression profiles triggered upon pathogen exposure in endemic versus non-endemic settings (**Figure 4**). Further analyses (by the employment of unsupervised ML algorithms) were performed to delineate gene expression profiles based on endemicity. For hybrid clustering (check method section) based on logFC values, the optimal number of clusters was pinned down to be six (based on the calculations of the “total with sum of square” values) (**Supplementary Figure S5**). Among the derived six clusters, cluster 2 was negatively associated with the *S. typhi* (Vietnam) cohort while cluster 4 was positively associated with this cohort. **Figure 5A** illustrates distinct gene expression patterns as observed in clusters 2 and 4, which distinguishes the *S. typhi* (Vietnam) cohort from the other three cohorts. Network construction and topological analysis of cluster 4 revealed ribosomal proteins (RPL22, RPS9, and RPS15) and genes associated with Hedgehog (JAG1, WNT2B, and ADAM17) signaling to be high-ranking hub genes as per the MCC and DMNC algorithm (**Figure 5B**). For cluster 2 (downregulated in the endemic cohort), the derived hub genes were mainly involved with growth factor receptor signaling (PTPN1, PTPN11, ERBB2, GRB2, FGF12, and PDGFRA), cell cycle signaling (WT1), and regulation of interferon signaling (SOCS1 and SOCS3). The findings of clustering analysis indicated upregulation of the Hedgehog signaling pathway and downregulation of growth factor receptor signaling to be specific attributes of the endemic cohort that distinguishes it from the other cohorts.

**Figure 6:**
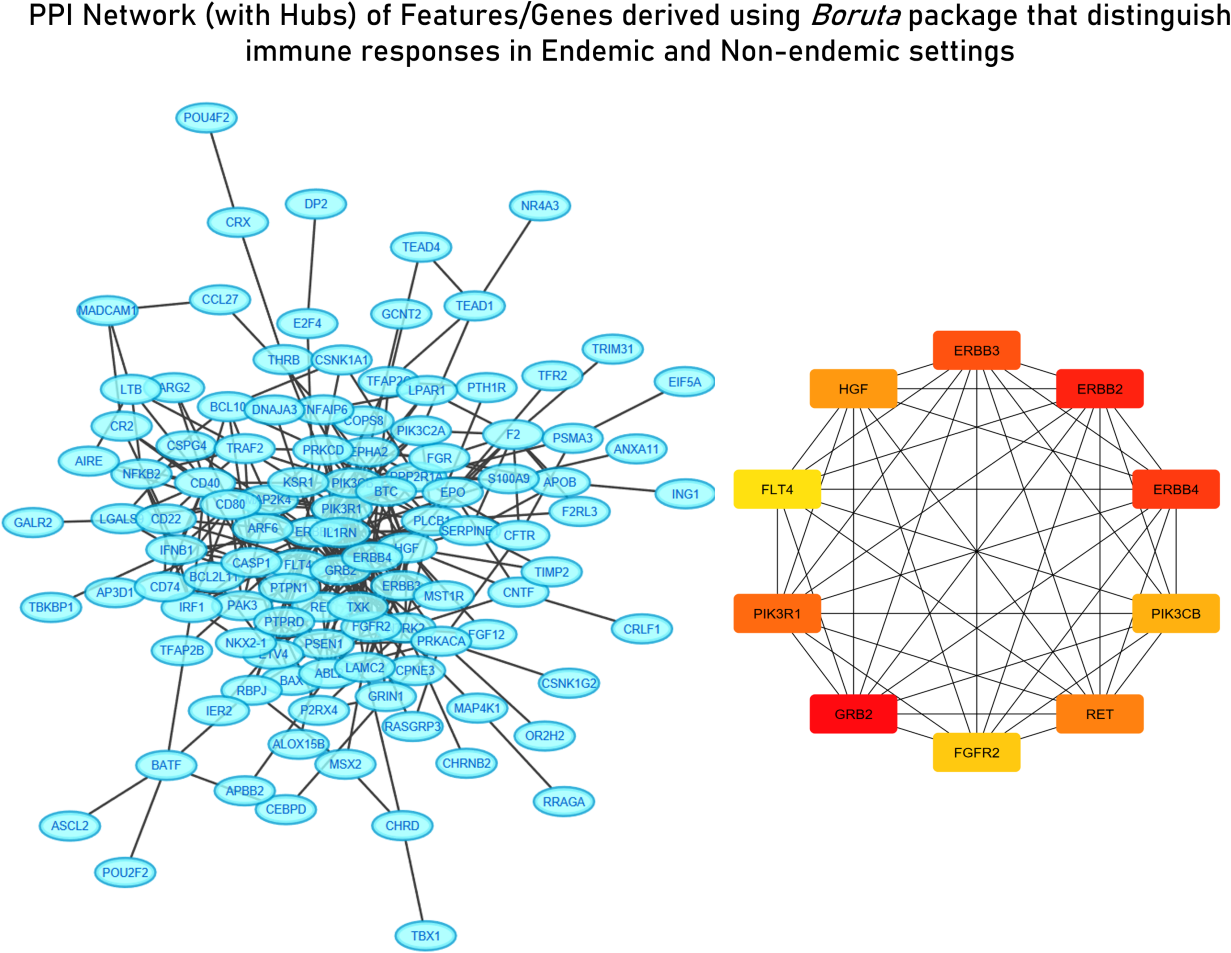
STRING network with derived hub genes of feature derived from the Random Forest algorithm distinguishing immune responses in endemic and non-endemic settings derived using the Boruta package.

For deriving more reliable features, Random Forest-based feature selection was used on the meta-dataset to derive highly influential determiners (features/genes) in characterizing host responses to enteric pathogens in endemic and non-endemic settings. Network construction and analysis of the derived features revealed hub genes associated with growth factor receptor and PI3K/Akt signaling (ERBB2, ERBB3, FGFR2, PIK3CB, PIK3R1, PIK3CD, and PTPN11) and genes associated with the cell cycle (CCND1 and RET) (**Figure 6**). All the three groups of features were characterized via functional enrichment analysis using the reactome database, and their key regulators were then retrieved from the TRRUST database (**Table 2**). Interestingly, the Random Forest-based feature selection again pointed out towards growth factor receptor signaling as an integral distinguishing feature of the endemic cohort compared to the non-endemic cohort, further validating the findings of the clustering analysis.

**Table 2:** Enriched reactome pathways derived using different methodologies specific for endemic settings along with their key regulators (FDR< 0.05, strength > 0.90, top 10).

Methodology	Enriched reactome pathways	Regulators
**Network Topological Analysis of DEGs**	•Signaling by FGFR3 fusions in cancer (**HSA-8853334**) •Signaling by PDGFRA transmembrane, juxta- membrane, and kinase domain mutants (**HSA-9673767**) •Activated NTRK2 signals through RAS (**HSA-9026519**) Signaling by FGFR4 in disease (**HSA-5655291**) •Constitutive signaling by overexpressed ERBB2 (**HSA-9634285**) Constitutive signaling by EGFRvIII (**HSA- 5637810**) •MET activates PI3K/AKT signaling (**HSA- 8851907**)	MYB, SP1
**Hybrid Clustering based on LogFC values (Cluster 2)**	•Regulation of IFNG signaling (**HSA-877312**) •Signaling by CSF3 (G-CSF) (**HSA-9674555**) •Spry regulation of FGF signaling (**HSA-1295596**) •Regulation of KIT signaling (**HSA-1433559**) •Inactivation of CSF3 (G-CSF) signaling (**HSA- 9705462**) •Regulation of IFNA/IFNB signaling (**HSA-912694**) •CTLA4 inhibitory signaling (**HSA-389513**) •Growth hormone receptor signaling (**HSA-982772**) •Signaling by PTK6 (**HSA-8848021**) •Signaling by SCF-KIT (**HSA-1433557**)	MYB, SP1, SP3, SMARCA4, HIF1A, ETS1, GLI1, CTTNB1, PAX2, STAT5B, ETS2, RELA, NFKB1, NR2C1, SP4, STAT1, YY1, AR, HOXA10, ATF3, DDIT3, GLI2, EP300, ELK1, KLF6, NR1H4, E2F4, ATF1, HDAC3, PGR, TCF4, HDAC1, TFAP2A, CTCF, **STAT3**, JUND, RUNX1, TP53, VDR, USF2, CEBPA, IRF1, BRCA1, GATA1, CEBPB, EGR1, CREB1, MYC
**Hybrid Clustering based on LogFC values (Cluster 4)**	•Hedgehog ligand biogenesis (**HSA-5358346**) •TP53 regulates transcription of cell death genes (**HSA-5633008**) •Release of Hh-Np from the secreting cell (**HSA- 5362798**) •Activation, translocation, and oligomerization of BAX (**HSA-114294**) •Nonsense mediated decay (NMD) independent of the Exon Junction Complex (EJC) (**HSA-975956**)	SP1, SMAD4, RELA, CTCF, ABL1, SNAI1, JUND, NR3C1, CREB5, E2F3, STAT5A, ZEB1, HIF1A, SNAI1, STAT1, FOSL2, BCL6, FOXO3, FOS, WT1, SOX9, SP3, FOXO1, NFKB1, PARP1, LEF1, CIITA, REST, ETS1, ATF, **STAT3**, JUN, EZH2, VDR, MYCN, BRCA1, SPI1, PPARG, HDAC1, ESR1, CREB1, AR, E2F1, TP53
**Features from Wrapper Algorithm with Random Forest**	•SHC1 events in ERBB2 signaling (**HAS-1250196**) •PI3K events in ERBB2 signaling (**HAS-1963642**) •ERBB2 activates PTK6 signaling (**HAS-8847993**) •MET activates PI3K/AKT signaling (**HAS- 8851907**) •Activated NTRK2 signals through PI3K (**HAS- 9028335**) •GRB7 events in ERBB2 signaling (**HSA-1306955**) •GRB2 events in ERBB2 signaling (**HSA-1963640**) •ERBB2 regulates cell motility (**HSA-6785631**) •CD28-dependent Vav1 pathway (**HSA-389359**)	RELA, NFKB1, SP1, FOXA1, STAT1, TFAP2A, AR, NCOS, TRERF1, CUX1, SP3, BTF2, TFAP2C, IRF7, HIF1A, CREB1, NR4A1, FOXA2, NFKBIA, PML, ELK1, CEBPB, ETV4, ATF1, SRF, SAMD4, YBX1, SMAD3, YY1, PPARA, TP53, USF2, IRF1, EP300, SPI1, USF1, PPARG1, STAT3, JUN. ESR1, ETS1, E2F1

Based on the findings of the two unsupervised machine learning algorithms, the negative regulation of components of the growth factor receptor signaling pathways and the positive regulation of the Hedgehog/WNT signaling pathway were determined to be associated with immune responses in endemic settings. To investigate further if these mediators can act as primary determiners of differences in immune responses between endemic and non-endemic settings, we used neural network-based classification (MLP classifier).

### 3.4 Identification of highly influential genes using ML-based classification

Machine learning-based classification was performed on hub genes derived in sections 3.2 and 3.3, which were categorized as being “responsive” or “housekeeping” genes using the HRT Atlas (https://housekeeping.unicamp.br/) (**Table 3**). The “responsive” genes were then evaluated for their potential to act as a classifier of immune responses for the endemic cohort compared to the non-endemic cohort using multiple supervised machine learning algorithms. Neural network-based classification algorithms were used for the analysis because of their documented compatibility to accommodate, analyze, and evaluate gene expression data (26).

**Table 3:** List of hub genes specific for the endemic cohort derived using different methodologies along with their corresponding functional roles and regulators (as identified from TRRUST database).

Source	Hub genes*	Biological process	Role	Key regulators
**Network** **Topological Analysis of DEGs**	**HRAS**	GO:0000165: MAPK cascade	Housekeeping	N/A
	**SOS1**	GO:0002260: Lymphocyte homeostasis	Housekeeping	N/A
	**KRAS**	GO:0000165: MAPK cascade	Housekeeping	N/A
	**SRC**	GO:0002376: Immune system processes	**Responsive**	SP1, TAF1
	**EGFR**	GO:0038134: ERBB2- *EGFR* signaling	**Responsive**	AR, BCL3 BRAC1, CREBBP, EGR1, ESR1, HDAC1/3, HOXB7, JUN, JUNB, KLF10, LRRFIP1, MTA1, NFKB1, NR3C2, PGR, PML, PPARG, RELA, SP1
	**MAPK1**	GO:0000165: MAPK cascade	Housekeeping	**N/A**
	**MAPK14**	GO:0000165: MAPK cascade	Housekeeping	N/A
	**PTK2**	GO:0001932: Regulation of protein phosphorylation	**Responsive**	N/A
	**UBB**	GO:0016567: Protein ubiquitination	Housekeeping	N/A
**Hybrid Clustering based on LogFC values (Cluster 2)**	**GRB2**	GO:0007173: EGFR signaling	**Responsive**	**N/A**
	**ERBB2**	GO:0004714: Transmembrane receptor protein tyrosine kinase activity	**Responsive**	AR, ATF, CREB1, DENND4A, ELF1, EP300, ETV4, FOXP3, GATA4, JUND, MYB, NCOA3, PAX2, PGR, PURA, SP1, TFAP2A, VDR, XRCC5, YBX1, YY1
	**PTPN11**	GO:0000077: DNA damage checkpoint signaling	Housekeeping	N/A
	**SOCS1**	GO:001817: Regulation of cytokine production	**Responsive**	GL1/2, HIF1A,IRF1, SP1, STAT3/6
	**PIK3CD**	GO:0002250: Adaptive immune response	**Responsive**	RUNX1
	**SOCS3**	GO:001817: Regulation of cytokine production GO:0000082:	**Responsive**	CEBPA, NFKB1, RELA, SP3, STAT1/3/4
	**CCND1**	G1/S transition mitotic cell cycle	Housekeeping	N/A
	**PDGFRA**	GO:0001775: Cell activation	Housekeeping	N/A
	**CSF3R**	HSA:9674555: Signaling by CSF3	**Responsive**	CEBPA, ETS1, MYB, SPI1
	**LCK**	HAS:389356: CD28 co-stimulation	**Responsive**	MYB
	**FGF13**	GO:0000165: MAPK cascade	Housekeeping	N/A
	**WT1**	HAS:9675108: Nervous system development	**Responsive**	CTCF, EP300, ETS1, GATA1/2, HDAC4/5, HOXA10, IFI6, MYB, NFKB1, PAX2/8, RELA, SP1, TFCP2
	**PHGDH**	GO0006541: Glutamine metabolic process GO:0033209:	**Responsive**	HOXA10, SP1
	**KRT18**	Tumor necrosis Factor-mediated signaling pathway	**Responsive**	BRCA1, CTBP1, SP1
	**PTPN1**	HAS:163615: PKA activation	Housekeeping	N/A
**Hybrid Clustering** **based on LogFC values** **(Cluster 4)**	**RPS16**	GO:0006364: rRNA processing	Housekeeping	N/A
	**RPL6**	Same as above	Housekeeping	N/A
	**RPS9**	Same as above	Housekeeping	N/A
	**RPL22**	Same as above	Housekeeping	N/A
	**RPS15**	Same as above	Housekeeping	N/A
	**ETF1**	GO:0006415: Translational Termination	Housekeeping	N/A
	**SOX2**	HAS-452271: Signaling by WNT	**Responsive**	ID4, KDM2A, POU5F1
	**FN1**	GO:0006953: Acute-phase response	**Responsive**	AR, ATF2, CEBPA, EGR1, KLF8, NFKB1, PARP1, RELA, SNAI1, SOX17, TWIST1/2
	**HSP90AA1**	GO:0002218: Activation of innate immune response	Housekeeping	N/A
	**EEF1D**	GO:0009299: Translational elongation	Housekeeping	N/A
	**WNT2B**	HAS:3238698: WNT ligand biogenesis and trafficking	**Responsive**	GLI2
	**JAG1**	HAS:2979096:NOTCH2 activation and transcriptional signal to the Nucleus	**Responsive**	KDM4C, PPARG, RUNX3, SNAI2
	**TLR6**		**Responsive**	HIF1A
**Features from Wrapper Algorithm with Random Forest**	**GRB2**	GO:0007173: EGFR signaling	**Responsive**	
	**ERBB2**	See above	**Responsive**	See above
	**ERBB4**	GO: 0006916: Apoptotic process	**Responsive**	WWP1
	**ERBB3**	GO:0007162: Negative regulation of cell adhesion	**Responsive**	AR, TWIST1/2, YBX1
	**PIK3R1**	GO:0002687: Positive regulation of leukocyte migration	**Responsive**	N/A
	**RET**	GO:0000165: MAPK cascade	**Responsive**	ESR1, FOXA1, SOX10, NKX2-1, TFAP2C
	**TXK**	GO0001819: Positive regulation of cytokine production	Housekeeping	N/A
	**MST1R**	GO:0002376: Immune system processes	Responsive	N/A

Functional roles identified from: https://housekeeping.unicamp.br/.

N/A, Not Available.

The performance of the classifiers was evaluated after the derivation of confusion matrices (based on the performed threefold classification). Based on accuracy and ROC, the genes were ranked based on their significance in differentiating immune responses in endemic and non-endemic settings grb2, an adaptor of tcr signalling was found to have the best performing score in classifying infected cohort from endemic and non-endemic setting (**Table 4**).

**Table 4:** MLP classification evaluation of the identified hub genes based on threefold classification.

Gene_LIST	Accuracy	Precision	Recall	*F*-measure	ROC area
**GRB2**	100%	1	1	1	1
**PIK3R1**	98.86%	1	0.952	0.976	0.973
**ERBB3**	97.72%	0.952	0.952	0.952	0.971
**ERBB4**	97.72%	0.952	0.952	0.952	0.999
**RET**	95.45%	0.947	0.857	0.9	0.925
**ERBB2**	94.31%	0.86	0.905	0.884	0.99
**TLR6**	92.04%	0.889	0.763	0.821	0.902
**SOX2**	90.90%	0.741	0.952	0.833	0.942
**EGFR**	89.77%	0.8	0.762	0.78	0.979
**PTK2**	88.63%	1	0.524	0.688	0.728
**SOCS1**	88.63%	0.824	0.667	0.737	0.841
**PIK3CD**	87.50%	0.917	0.524	0.667	0.781
**PHGDH**	85.22%	0.682	0.714	0.698	0.84
**CSF3R**	78.40%	0.583	0.333	0.424	0.768
**KRT18**	77.27%	0.667	0.095	0.167	0.569
**FN1**	77.27%	0.52	0.619	0.565	0.741
**WNT2B**	76.13%	NA	NA	NA	0.599
**JAG1**	76.13%	NA	NA	NA	0.482
**SOCS3**	76.13%	0.5	0.238	0.323	0.841
**LCK**	75%	0	0	0	0.385
**WT1**	72.72%	0.385	0.238	0.294	0.731

Model construction and evaluation were performed using the WEKA software.

N/A, Not Available.

#### 3.4.1 Validation of GRB2 as a classifier

To validate GRB2 as a high-performing classifier, two other machine learning algorithms were built to construct the classification model, where, again, GRB2 was classified with high accuracy (**Supplementary Figure S7**). To validate GRB2 suppression at the acute stage upon vaccination, the ImmuneSpace database was screened for trials that have reported GRB2 downregulation in the first 7 days after immunization. The findings of the survey are tabulated in **Supplementary Table S7** where we found four clinical trials with indications of GRB2 suppresion at the acute stage post immunisation.

### 3.5 Correlation between TCR and Hedgehog/NOTCH signaling pathways

**Figure 7:**
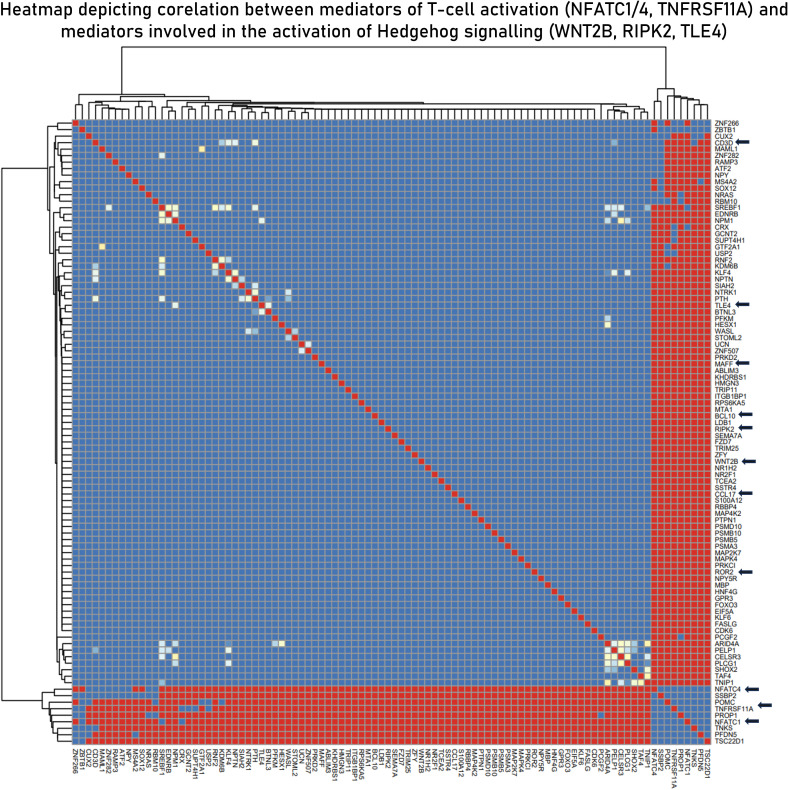
Curated submodule derived from module 3 correlation module derived from the *EBModules* package that shows the positive associations of positive regulators of T-cell activation with mediators of the Hedgehog signaling pathways. Here, red bricks indicate a high correlation coefficient of 1, blue bricks indicate a correlation coefficient of 0, and yellow bricks indicate intermediate correlation coefficient.

Based on the hypothesis generated in sections 3.2, 3.3 and 3.4, to derive the relationship between the two signaling pathways (TCR and Hedgehog), correlation studies were performed. A total of 20 correlation modules (group of genes) were identified in the integrated datasets. These modules were characterized using functional enrichment analysis and were filtered using the master list (**Supplementary File 2**) to derive immunologically relevant submodules (**Supplementary Table S7**). We found the curated submodule retrieved from module 3 to contain components of both TCR signaling (NFATC4 and NFATC1) and Hedgehog signaling (WNT2B, TLE4, MAFF, and ROR2) and to be highly correlated. NFATC1/4 are transcription factors associated with activated T cells, and their positive correlation with the components of the Hedgehog signaling pathway indicates activation of the latter in activated T cells. We also found CCL17, a known chemotactic agent of T cells, to be correlated with NFATC1/4 transcription factors (**Figure 7**).

### 3.6 Multivariant regression models to determine predictors of highly influential genes

**Figure 8:**
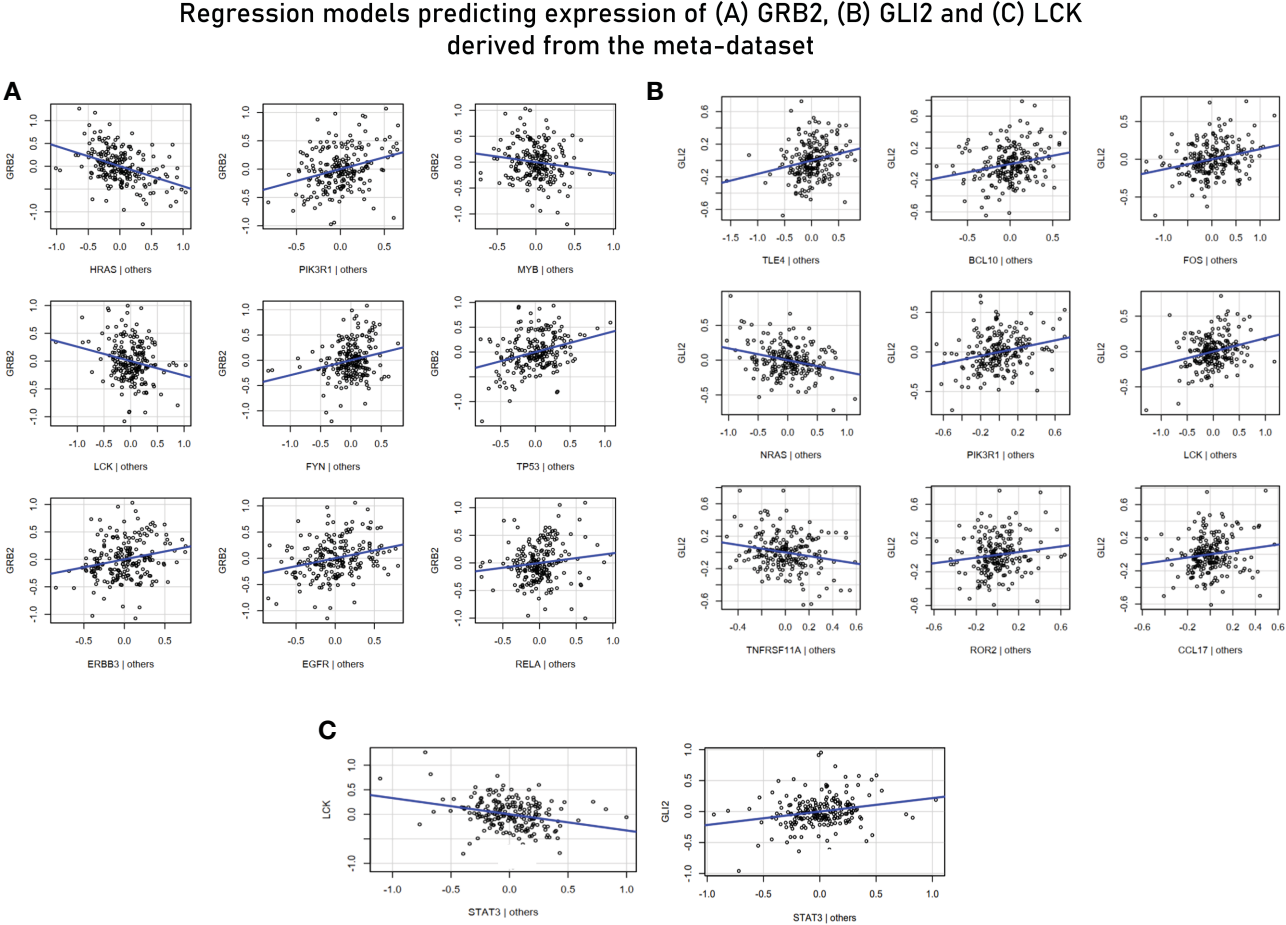
**(A)** Multivariant regression model for GRB2 (*R* ^2^ = 0.76). While the rest of the predictors showed a positive association with GRB2, HRAS, MYB (TF for LCK), and LCK demonstrated a negative association. **(B)** Multivariant regression model for GLI2 (TF for WNT2B) (*R* ^2^ = 0.70). The model demonstrated positive associations of GL2 with key mediators of TCR signaling: BCL10, PIK3R1, LCK, and TNFRSF11A. **(C)** Regression model predicting the association of LCK and GLI2 with STAT3.

For MVR analysis, housekeeping genes identified as highly influential genes in sections 3.2, 3.3 and 3.5 were taken as predictor variables and genes associated with effector functions (or are “responsive” to external stimuli) were taken ahead for the analysis as the response variables—GRB2, LCK, GLI (TF for WNT2B receptor) (**Table 3**). Potential predictor variables for these four genes were also retrieved from correlation modules in section 3.5. The MVR model for GRB2 yielded a high *R* ^2^ value of 0.7616 and its components/predictors were retrieved from network cluster 1 (**Supplementary Figure S2**). While other predictors showed a positive association with the target gene GRB2, LCK, MYB (TF of LCK), and HRAS showed a strong negative relation and were upregulated in the endemic cohort while the GRB2 was downregulated. The multiple regression model against GLI2 (a transcription factor for WNT2B) involving TLE4, BCL10, FOS, NRAS, PIK3R1, LCK, TNFRSF11A, ROR2, and CCL17 yielded an *R* ^2^ value of 0.708, and these predictors were retrieved from correlation module 3 (**Figure 8**). To investigate if there are common transcription factors that regulate both TCR signaling and the Hedgehog signaling pathway, univariate regression studies were performed for the mediators of the two signaling pathways. Although we did not find any single transcription factor as a common regulator of GRB2 and other mediators of Hedgehog signaling, we did find STAT3 to be negatively associated with LCK (another prominent adaptor in TCR signaling) and to be positively associated with GLI2 expression. Based on these findings, we inferred STAT3 to be a balancing transcription factor that, on one hand, regulates TCR signaling while promoting the induction of Hedgehog signaling on the other hand (**Figure 8C**).

### 3.7 Retrieved gene regulatory modules

Gene regulatory module 1 (GRM1) was inferred from the GRB2 multivariant model wherein, based on literature, the central role of GRB2 in TCR signaling was identified and key regulatory elements found in this study were integrated (**Supplementary Figure S2**). Several relevant findings from the obtained results were considered for module construction: (i) Genes involved in TCR signaling were both up- and downregulated upon KEGG pathway enrichment analysis (GRB2 being downregulated) (**Supplementary Figures S3** and **S4**), (ii) downregulation of a cluster of genes (with GRB2 being a hub gene) involved in growth factor receptor signaling (**Figure 5** and **Table 2**), (iii) GRB2 being one of the hub genes in the network obtained through Random Forest-based feature selection (**Figure 6**), and (iv) GRB2 performing perfectly as a classifier of immune responses in endemic and non-endemic settings (**Table 4** and **Supplementary Figure S7**). Based on these findings, we hypothesize that GRB2 might play an integral role in downregulating growth factor receptor signaling and in negatively regulating downstream TCR signaling in the endemic cohort. Moreover, the MVR model derived for GRB2 (through a combinatorial approach) suggests that while PIK3R1, TP53, FYN, and RELA (from the model in **Figure 8**), which act downstream of TCR signaling (**Supplementary Figures S3** and **S4**), would be affected by GRB2 suppression, other downstream mediators might actually act as negative regulators (HRAS, MYB, and LCK).

**Figure 9:**
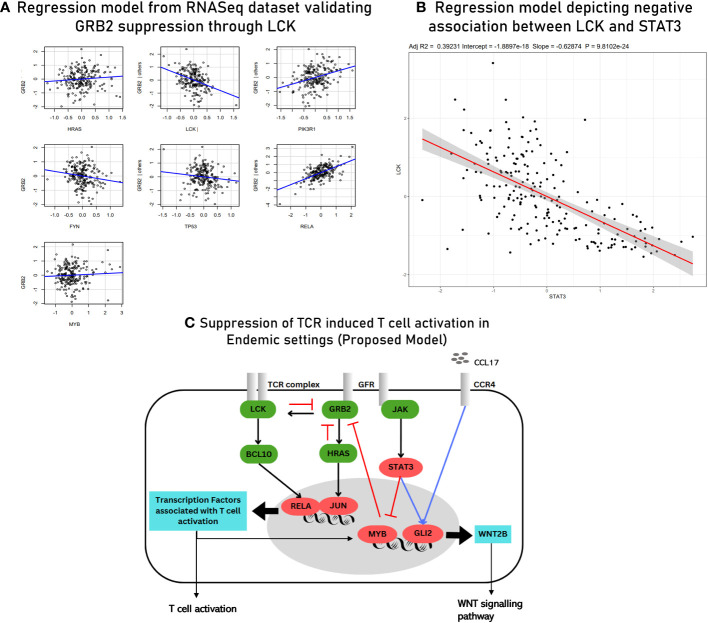
**(A)** Multivariant regression model for GRB2 suppression with *R* ^2^ = 0.54 derived from RNASeq data (validation). **(B)** Scatter plot depicting a negative association between LCK and STAT3 derived using RNASeq data (validation). **(C)** Proposed model of TCR signaling upon acute infection in endemic settings. The known/established regulatory associations in TCR signaling are depicted with black arrows. The negative regulation of GRB2 (depicted with red inhibitory arrows) is inferred from gene regulatory module 1. The induction of GLI2 by the transcription factors associated with activated T cells and through CCL17-based signaling (blue arrow) is inferred from gene regulatory module 2. STAT3-mediated inhibition of MYB (transcription factor for LCK) (red arrow) and the positive regulation of GLI2 (blue arrow) are inferred from the findings of gene regulatory module 3. The three gene regulatory modules are described in detail in the **Supplementary File 8**.

The second gene regulatory module (GRM2) was inferred using the MVR model for GLI2. Interactions of GLI2 with transcription factors and other mediators of TCR signaling and extracellular mediators involved in chemotaxis of lymphocytes were closely studied (**Table 3**). Through GRM2, we propose Hedgehog signaling pathways as primary differentiators of matured lymphocytes as compared to lymphocytes being freshly induced. Based on the results obtained from hybrid clustering (**Figure 5**), we propose them to be closely involved in T-cell function in endemic settings upon infection. The third gene regulatory module (GRM3) was specially retrieved based on the regulatory dynamics observed for STAT3 in two different regression models (**Figure 8C**). Based on our observations, we propose STAT3 as a primary determinant responsible for state switching of T cells upon infection by, on one hand, directly/indirectly negatively regulating TCR induction and, on the other hand, nudging towards Hedgehog signaling. Regulatory modules of GRB2 suppression and the negative association between STAT3 and LCK as derived from the meta-analysis were validated via the RNASeq dataset using a regression model (with an *R* ^2^ value of 0.5441) (**Figure 9**). The culmination of the key findings (which distinguish acute immune responses in endemic and non-endemic settings) from the study is illustrated in the form of a model in **Figure 9**. For the development of this model, established molecular interactions in TCR signaling were retrieved from literature (33).

**Supplementary File 7** provides a more detailed rationale used for the construction of gene regulatory modules while **Supplementary Figure S6** provides an illustrative summary of the entire study.

## 4 Discussion

Enteric vaccines have been reported to show low efficacy in regions that are highly endemic to pathogens (4–6). Apart from enteric infections, vaccines against other infectious diseases have also shown similar tendencies. For example, in a study, the YF-17D, the yellow fever vaccine, showed low vaccine efficacy in an African cohort, which the author attributed to an “activated” microenvironment in the study population—including “differentiated T and B cells and pro-inflammatory cytokine secreting monocytes” (34). On similar lines, recently, it has been observed that infection with SARS-CoV-2 with its different variants generates cross-reactive T cells, which are not necessarily protective, but had a direct impact on vaccine effectiveness (35, 36). These findings imply that pre-existing immunity against specific pathogens can have a direct impact on immune responses to subsequent immunization attempts. With SARS-CoV-2 becoming endemic worldwide, the design and development of the next generation of COVID-19 vaccines and advanced vaccines against other endemic infections would require keen consideration to pre-existing protective/semi-protective/non-protective immunity against these pathogens in the target population.

Hence, understanding the immunological dynamics of re-infection in general and the possible impact of immunization in a chronically exposed population becomes absolutely essential for the development of future vaccines that are region- and population-specific (15, 37). In this regard, several studies have investigated immune responses against malaria and other helminth infection in a previously exposed population. One of these studies reported acute upregulation of co-stimulatory molecules (like CD40, CD80, and CD86) upon stimulation of dendritic cells in experienced (38). Another study indicated the important role of γδ T cells in secondary immune responses to malaria in endemic settings (39). Moreover, an immunomodulatory effect of chronic exposure to parasitic infections has also been reported against parasitic infections (40). Such studies are still lagging behind for enteric infections in endemic settings. Using an intensive systems and computational pipeline, we have designated molecular signatures and transcriptional regulatory networks that delineate acute immune responses in endemic settings in comparison to those induced in non-endemic settings, taking enteric infections as a case study. Importantly, we show that (i) there is a negative feedback regulation of downstream signaling pathway associated with T-cell activation through GRB2 downregulation (GRM1), (ii) WNT receptor expression in activated T cells is under the influence of CCL17 (GRM2), and (iii) STAT3 mediated the state change of activated T cells through the upregulation of WNT receptor (GRM3).

To elaborate on the first regulatory module (GRM1), GRB2 is an adaptor molecule assembled and recruited near the intracellular chains of growth factor receptors involved in the activation of RAS, which unleashes the downstream signaling pathways. GRB2 also plays an essential role in TCR signaling by propagating activation/proliferation signals intracellularly after synapse formation of the TCR complex with the peptide–MHC complex through the activation of MAPK signaling pathway. Upon TCR/co-receptor stimulation of LCK, an SRC family tyrosine kinase,* gets activated and, through a short series of phosphorylation, recruits ZAP-70, which, in turn, facilitates the assembly of downstream scaffolds that includes the Linker Activator of T-cells (LAT). LAT provides a platform for GRB2 (and for other adaptor molecules) assembly where GRB2 relays the received signals through RAS activation (41). Because of its early involvement in signaling events, GRB2 has been designated as a rate-limiting and essential component of the TCR-induced MAPK/ERK signaling pathway, which is essential for lymphocyte selection, proliferation, and differentiation (42–44).

Owing to the constitutive and ubiquitous nature of the MAPK pathway and risk associated with its overexpression, several negative regulatory circuits have evolved throughout the signaling pathway downstream of TCR activation (45). Broadly, there are two channels of negative regulation that involve the phosphorylation-based functional inactivation of upstream mediators by activated ERK and, secondly, the transcriptional regulation of upstream mediators. In terms of GRB2 suppression, phosphorylation of LAT, which leads to its disassociation with GRB2, has been previously reported, which is an example of the former, and induction of SPRY protein (through ERK pathway activation) that binds and disables GRB2 action can be considered as an example of the latter (41, 46). Although post-translational regulation of GRB2 is well documented (46, 47), transcriptional regulation of GRB2 expression remains quite elusive in the literature.

Our study, particularly MVR analysis focusing on GRB2 expression using the gene expression dataset, indicates that high expression levels of HRAS, MYB (downstream mediators of growth factor receptor signaling), and LCK (adaptor for the TCR receptor) negatively affect GRB2 expression upon perturbation (antigenic exposure), which might negatively impact T-cell activation and proliferation. This observation is further validated by the fact that GRB2 was peculiarly downregulated at the acute stage of infection in an endemic setting and the fact that the TCR signaling pathway was also seen to be downregulated in this endemic cohort (**Supplementary Figure 8**). The molecular and transcriptional mechanism for suppression of GRB2 expression needs further investigation. Although MIR200a and microRNA have been reported to suppress the expression of GRB2, consequently negatively regulating the MAPK signaling pathway (48), its relevance in this particular setting is not known.

To further explore if the described phenomenon occurs upon vaccination as well, investigation of GRB2 expression levels in other vaccine clinical trials in the ImmuneSpace database was conducted. We found that clinical trials with ImmunPort accession IDs SDY299, SDY1328, SDY1276, and SDY180 (out of 47 studies reporting GRB2 expression levels) also report GRB2 downregulation at early time points of vaccination (**Supplementary Table S7**), validating GRB2 suppression as an acute immunomodulatory response in certain conditions. Gene expression datasets (post vaccination) from endemic settings were not available in the ImmuneSpace database (**Supplementary Figure S8**).

The outcomes of our analysis specifically might have profound implications in the vaccine design and development of endemicity/region-specific vaccines as it would provide explanation to previously ambiguous vaccine trial outcomes where unexpectedly suboptimal T-cell responses were observed (as discussed above). Importantly, as baseline-heightened immunological profile in the endemic cohorts is very well documented, we hypothesize that further perturbation/exposure/attack of pathogen might push TCR signaling into an auto-regulatory loop. This would imply that suboptimal vaccine efficacy observed in these regions would be the inherent characteristic of the vaccinees, and hence, increasing the dosage of a vaccine or using high adjuvanticity might not have the expected result and might actually disrupt the biological “sea-saw” or balance put in place to check for immune hyperactivity or even autoimmunity. This is worth considering particularly because several autoinflammatory and autoimmune diseases have been attributed to GRB2-linked molecular assemblies (41, 49). In the same line, in mice, it has been demonstrated that GRB2-induced MAPK/Erk signaling pathway might switch to hyperactivity if not negatively regulated by LCK (50) (negative association of LCK and GRB2 was demonstrated through our analysis) (**Figures 8** and **9**).

While GRB2 suppression solely would have indicated a regulatory immune response to infection in these settings, the observed GRM2 indicates a more multidimensional effector function of T cells. Overall, these findings suggest a biphasic transformative nature of T cells, which is dependent on the pathogenic load of the environment. In this regard, we propose STAT3 to be a key determiner of biphasic T-cell function in endemic settings based on its negative association with LCK expression and positive association with GLI2 (transcription factor for WNT2B receptor expression). Our findings are validated by the fact that STAT3 has been reported to dampen immune responses, which, in this case, can be a result of frequent exposure to enteric pathogens in pathogen-prevalent regions. STAT3 has also been reported to promote the activation of regulatory T-cell responses (51). Besides this, a strong indication of the WNT signaling pathway being involved in immune responses in endemic settings is an intriguing finding. Recently, WNT signaling has been reported to be activated in the local mucosa in subjects affected by environmental enteropathy, which is prominent in regions with endemicity of enteric infections (52). WNT signaling pathways have been reported to play an integral role in the differentiation and functioning of mature T cells particularly in the context of cell-to-cell communication and in cell migration/homing (53, 54). Given this, activation of these signaling pathways could mediate the induction of regulatory T cells (differentiation) as an immunomodulatory response to re-infection. These signaling pathways, especially the WNT signaling pathway, can also be involved in T-cell trafficking towards infected mucosa under the influence of activated leukocytes and, resultantly, cytokine secretion. Through our work, we also established positive associations between the induction of these pathways and the chemokine ligand CCL17, which is an established lymphocyte chemoattractant (GRM2) (55, 56).

Although the robust computational pipeline provides novel insights into the key molecular mechanisms that might be peculiar to endemic settings, the study is restricted by the sample size secured for the endemic population due to the unavailability/inaccessibility of immune response-linked gene expression datasets from these settings even after the systemic screening of public repositories. Another major limitation of the study is the loss of genes to a mere 6,543 genes in the meta-dataset, which could be considered as a “cost-of-merger” of heterogeneous gene expression datasets. We suspect that, like GRB2, we might come across other key molecular mediators that play an essential role in distinguishing immune responses in endemic and non-endemic populations that can only be uncovered by multicohort studies (from endemic and non-endemic settings) where pre- and post-infection/vaccination RNASeq data are retrieved for all the study groups.

Despite the mentioned limitations, in conclusion, through a novel methodical analytical pipeline, we demonstrate that gene expression datasets provide an unprecedented opportunity to understand variations in gene regulatory modules involved in immune responses to pathogens in different environmental settings (with a different pathogenic load). We used an amalgamation of systems (in the form of STRING networks) and advanced computational approaches (hybrid clustering, wrapper method for feature selection, MLP classification, correlation, and MVR analysis) to delineate immune responses specific to the endemic cohort of the study. Based on the findings of the study, we propose that perhaps the basal immune system and subsequent post-infection/vaccination immune responses diverge upon varying levels of previous exposures. Consequently, detailed insight into the reasons and principles behind these divergences should form the basis for the design and development of the “next-gen” precise vaccines. We put forward acute GRB2 suppression as a divergent (immunomodulatory) path the immune system evolves to take in endemic settings as one of the divergent paths the immune system evolves to take. While these observations are specific for *S. typhi* (intracellular bacterial) infection that attacks the enteric mucosa, further studies that look into the induction of the discussed regulatory molecules in other mucosal infections (possibly other enteric infections) can be an exciting start towards the development of endemicity-specific vaccines. From a global health standpoint, these studies should also include infections induced in the lung mucosa because of seasonal or perennial prevalence by pathogens like the influenza virus and quite recently by SARS CoV-2.

## Data availability statement

Publicly available datasets were analyzed in this study. This data can be found here: GSE7000, GSE112958, GSE95104, GSE2729, GSE69529.

## Ethics statement

Ethical approval was not required for the study involving humans in accordance with the local legislation and institutional requirements. Written informed consent to participate in this study was not required from the participants or the participants’ legal guardians/next of kin in accordance with the national legislation and the institutional requirements.

## Author contributions

AN: Conceptualization, Data curation, Formal analysis, Investigation, Methodology, Visualization, Writing – original draft. SL: Conceptualization, Methodology, Supervision, Validation, Writing – review & editing.
