# Supplementary material for: Systems and computational analysis of gene expression datasets reveals GRB-2 suppression as an acute immunomodulatory response against enteric infections in endemic settings
Source: Front Immunol. 2024 Feb 16;15:1285785. doi: 10.3389/fimmu.2024.1285785 (PMC10906661; doi:10.3389/fimmu.2024.1285785)
Supplement: Supplementary file 7 [file DataSheet_7.docx]

**Supplementary File 8**

**Retrieval of Gene Regulatory Modules (GRMs)**

Through all the three pipelines, Gene Regulatory Modules were screened out through pathway enrichment analysis and regression analysis. The associations between each of the genes/pathways in the cluster/modules/ feature group were determined using regression analysis. Key regulators of each of the cluster/modules/ feature group were retrieved using the TRRUST database.

**Gene Regulatory Module I (GRMI):**

***Negative Feedback Regulation of TCR signalling***

GRM1 was again identified as a network cluster from the *S. typhi* (Vietnam) cohort. The cluster was enriched in downstream signalling pathways of growth factor receptors (ERBB2/EGFR) and included receptor adaptor molecules (GRB2) and cell cycle associated molecules (HRAS, RHOA, PTPN11) (**Table 4**). Although not associated with any enriched pathway LCK and FYN, known mediators of TCR signalling upon activation, were also present in this cluster. Association between LCK/FYN with mediators involved in growth factor receptor signalling were studied using regression analysis (**Figure 7(II))**. We found a negative feedback loop leading to negative regulation of GRB2 expression (and eventual signalling) which was mediated by HRAS and LCK and its regulators. GRM2 is illustrated in **Figure 9 (II)**.

**Gene Regulatory Module II (GRM2):**

***WNT and NOTCH expression in activated T cells***

GRM2 was retrieved from common correlation modules (Pipeline 2) among the four datasets. Modules heavily enriched with immunologically relevant genes were taken further for the analysis – (Module 12, 20, 5, 9, 10 and 3) (**Table 5**). These modules were further screened to excavate novel associations between enriched pathways and co-related genes. As a result, we found that Module 3,5 and Module 9,10 uncovered unique co-relation of pathways involved in TCR/BCR activation and the ones involved in NOTCH/WNT signalling (**Table S5**). The genes involved were further analysed using regression analysis (**Figure 7(III))** to reveal a synergic role of TCR signalling and simultaneous stimulation by CCL17/CCL16 in inducing expression of WNT and NOTCH signalling receptors in lymphocytes. GRM3 is illustrated in **Figure 9 (III)**.

**Gene Regulatory Module III (GRM3)**

***STAT1/STAT3 mediated state-switch in T cells***

Pipeline 3 revealed specific transcriptional profiles in endemic and non-endemic settings. While cluster 4 (from hybrid clustering) was enriched with hedgehog associated pathways cluster 2 was enriched in signalling pathways linked with lymphocyte activation and stimulation (**Table 6**). While cluster 4 was positively associated with the endemic cohort, cluster 2 was negatively associated with it. To further elaborate on the regulatory framework which predisposes a lymphocyte for activation or for the expression of WNT/NOTCH receptor (and eventual signalling), the four feature groups derived from pipeline 3 (along with regulators) were analysed. LCK expression was chosen as a marker for T cell activation signalling, while GLI2 expression was chosen as a marker of WNT signalling. Out of all the regulators we found STAT3 to be important precursive regulators of NOTCH signalling and TCR signalling respectively (**Figure 8).** GRM5 is illustrated in **Figure 9 (V)**.

Results of multivariant regression studies (with R2 values) conducted for statistical validation of associations are presented as supplementary figures (**Figure S12-S17**).

**References**

Kryštof, V., Baumli, S., & Fürst, R. (2012). Perspective of Cyclin-dependent kinase 9 (CDK9) as a Drug Target. *Current Pharmaceutical Design*, *18*(20), 2883. https://doi.org/10.2174/138161212800672750

Shen, Y. li, Wang, Y. mao, Zhang, Y. xin, Ma, S. jie, Yang, L. he, Zhao, C. guang, & Huang, X. ying. (2021). Targeting cyclin-dependent kinase 9 in cancer therapy. *Acta Pharmacologica Sinica 2021 43:7*, *43*(7), 1633–1645. https://doi.org/10.1038/s41401-021-00796-0
