# Supplementary material for: Systems and computational analysis of gene expression datasets reveals GRB-2 suppression as an acute immunomodulatory response against enteric infections in endemic settings
Source: Front Immunol. 2024 Feb 16;15:1285785. doi: 10.3389/fimmu.2024.1285785 (PMC10906661; doi:10.3389/fimmu.2024.1285785)
Supplement: Supplementary file 8 [file Presentation_1.pptx]

## Slide 1
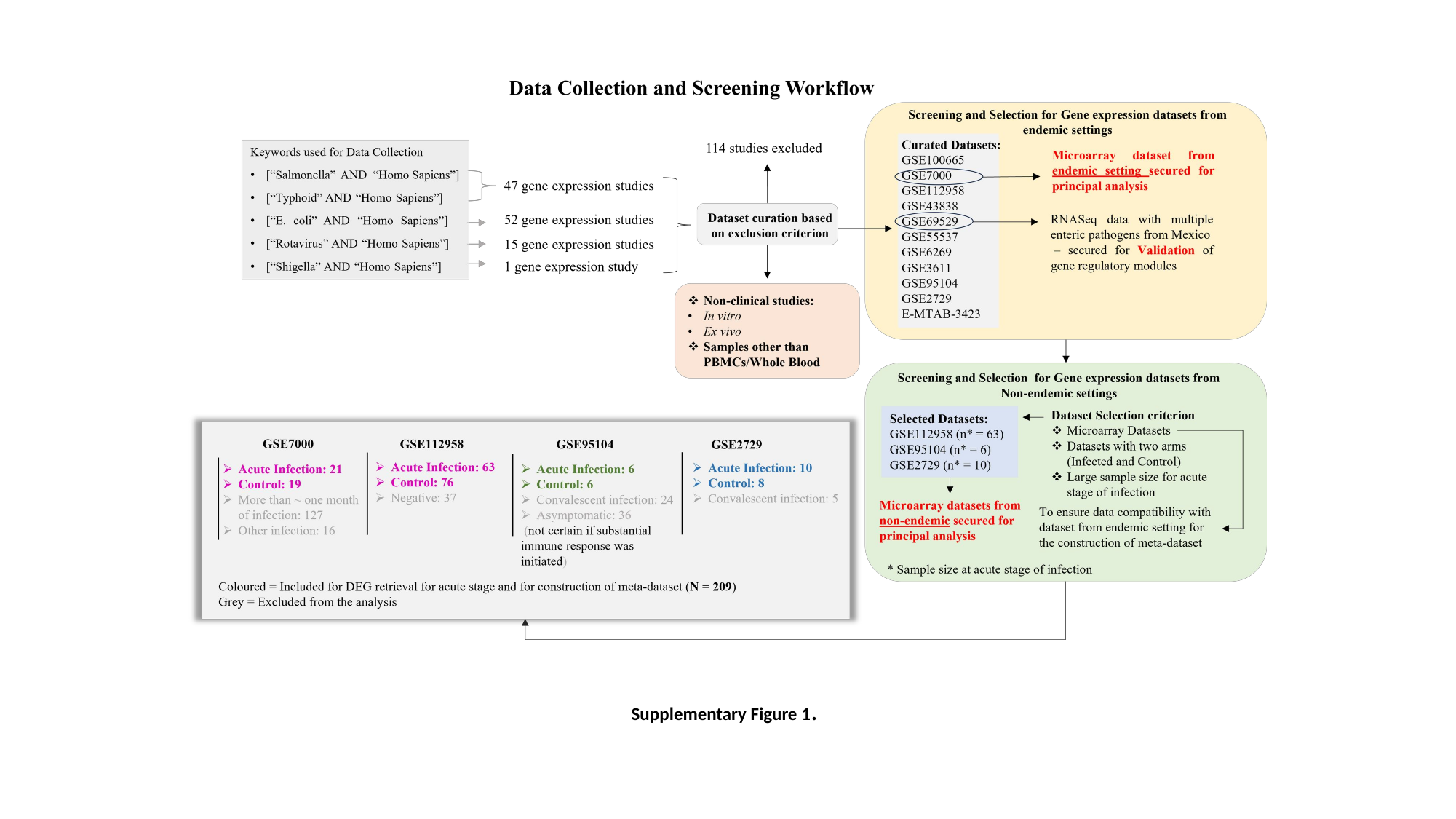

Supplementary Figure 1.

## Slide 2
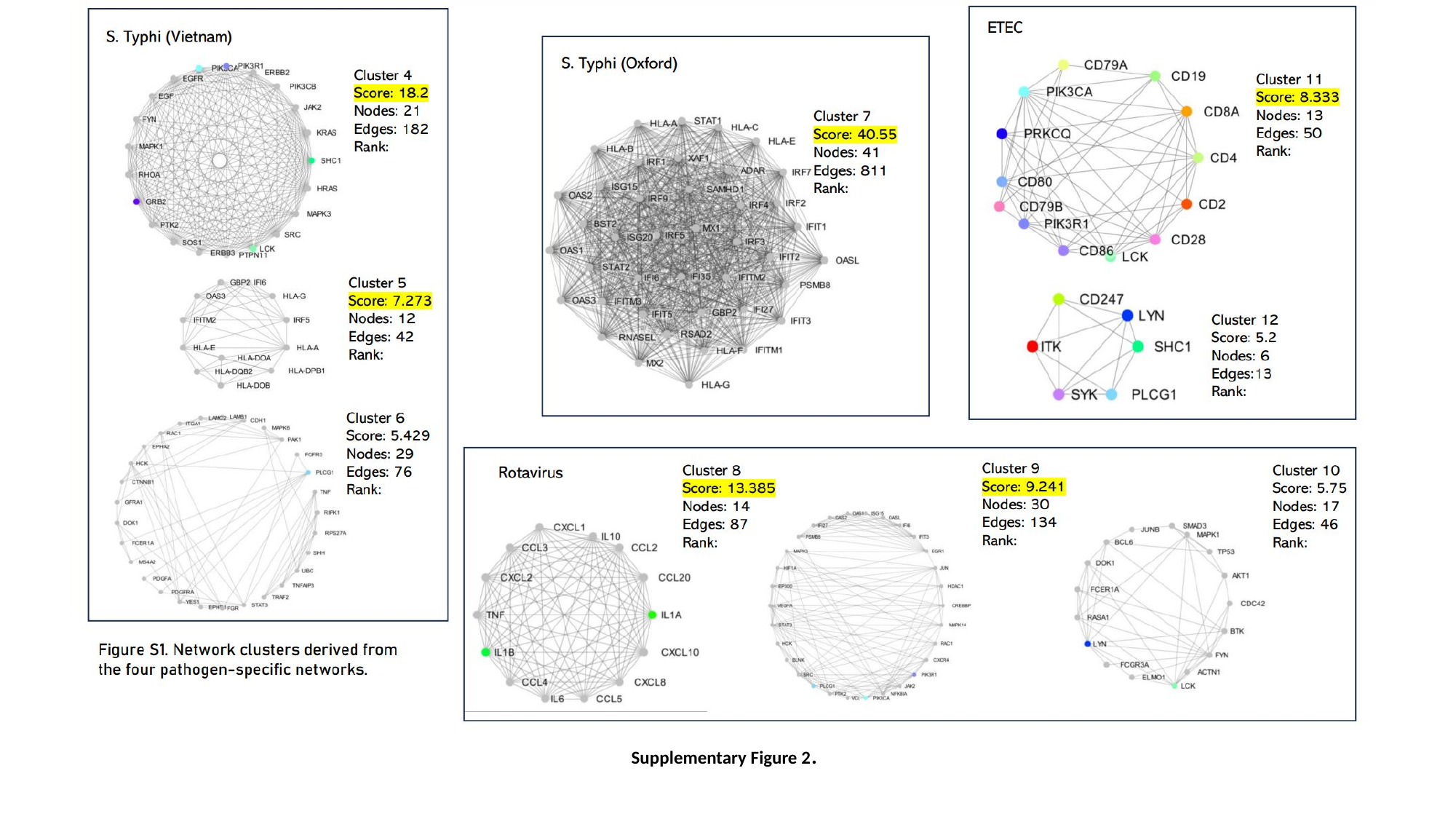

Supplementary Figure 2.

## Slide 3
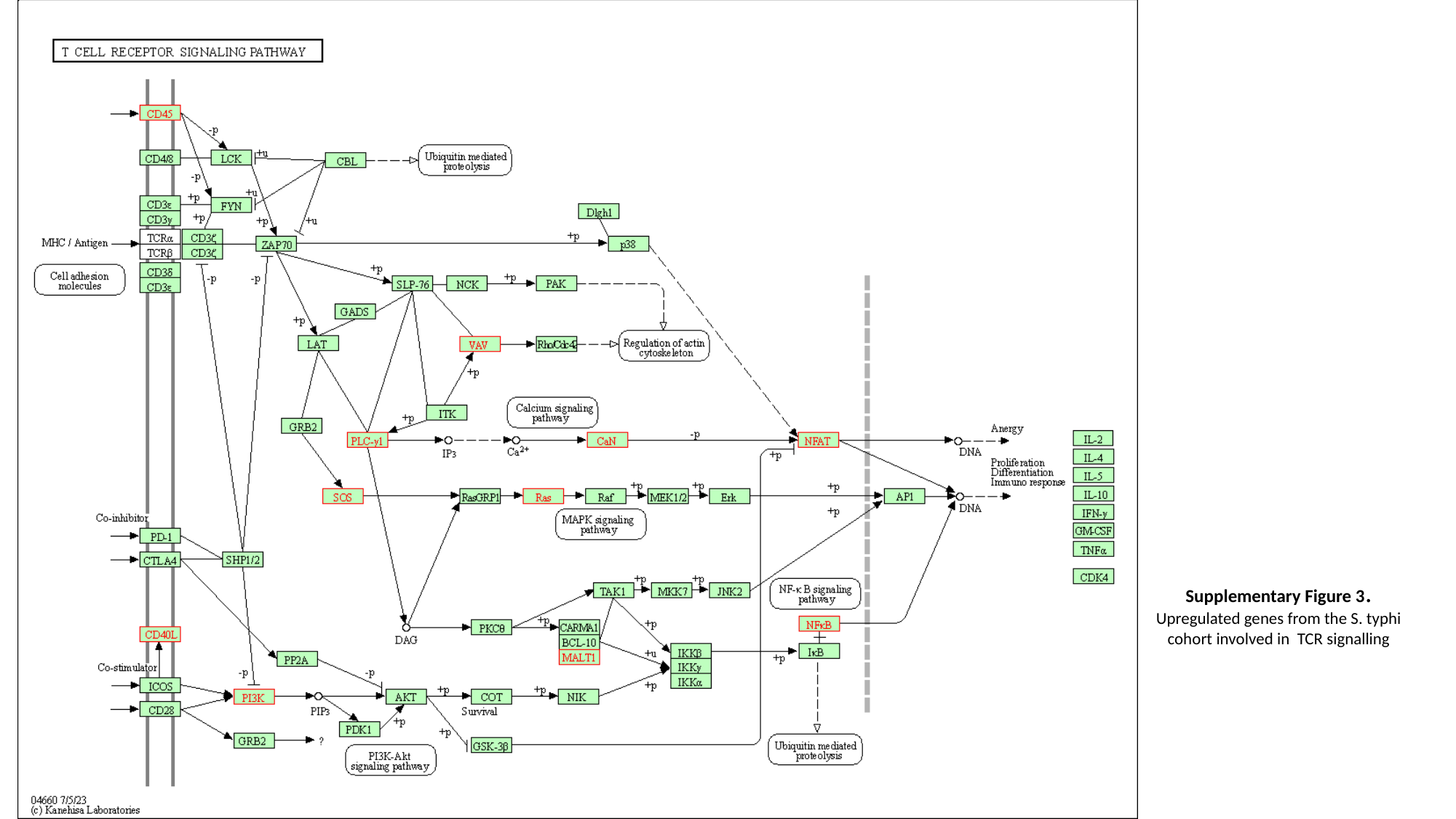

Supplementary Figure 3. Upregulated genes from the S. typhi cohort involved in TCR signalling

## Slide 4
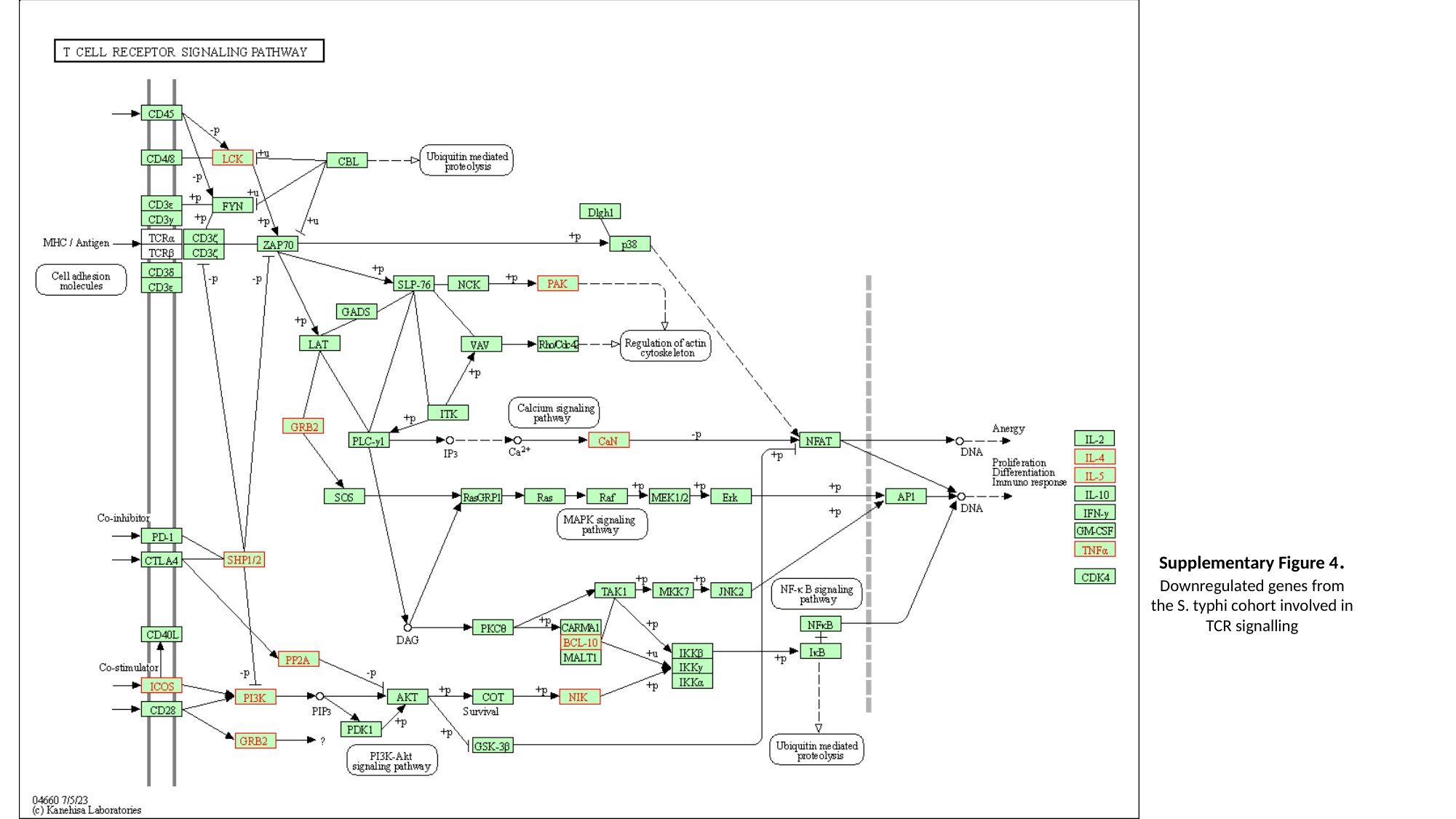

Supplementary Figure 4.
Downregulated genes from the S. typhi cohort involved in TCR signalling

## Slide 5
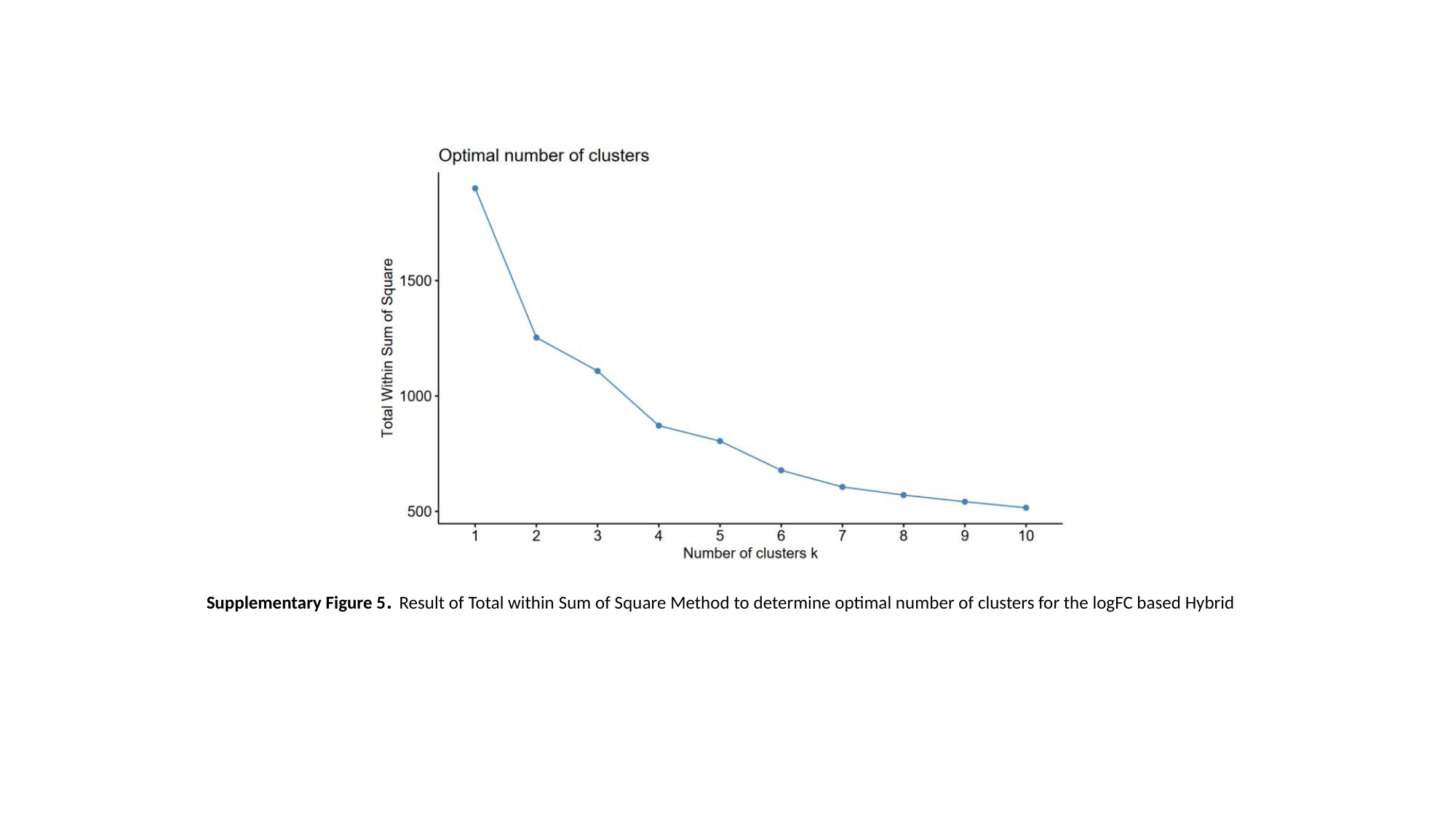

Supplementary Figure 5. Result of Total within Sum of Square Method to determine optimal number of clusters for the logFC based Hybrid

## Slide 6
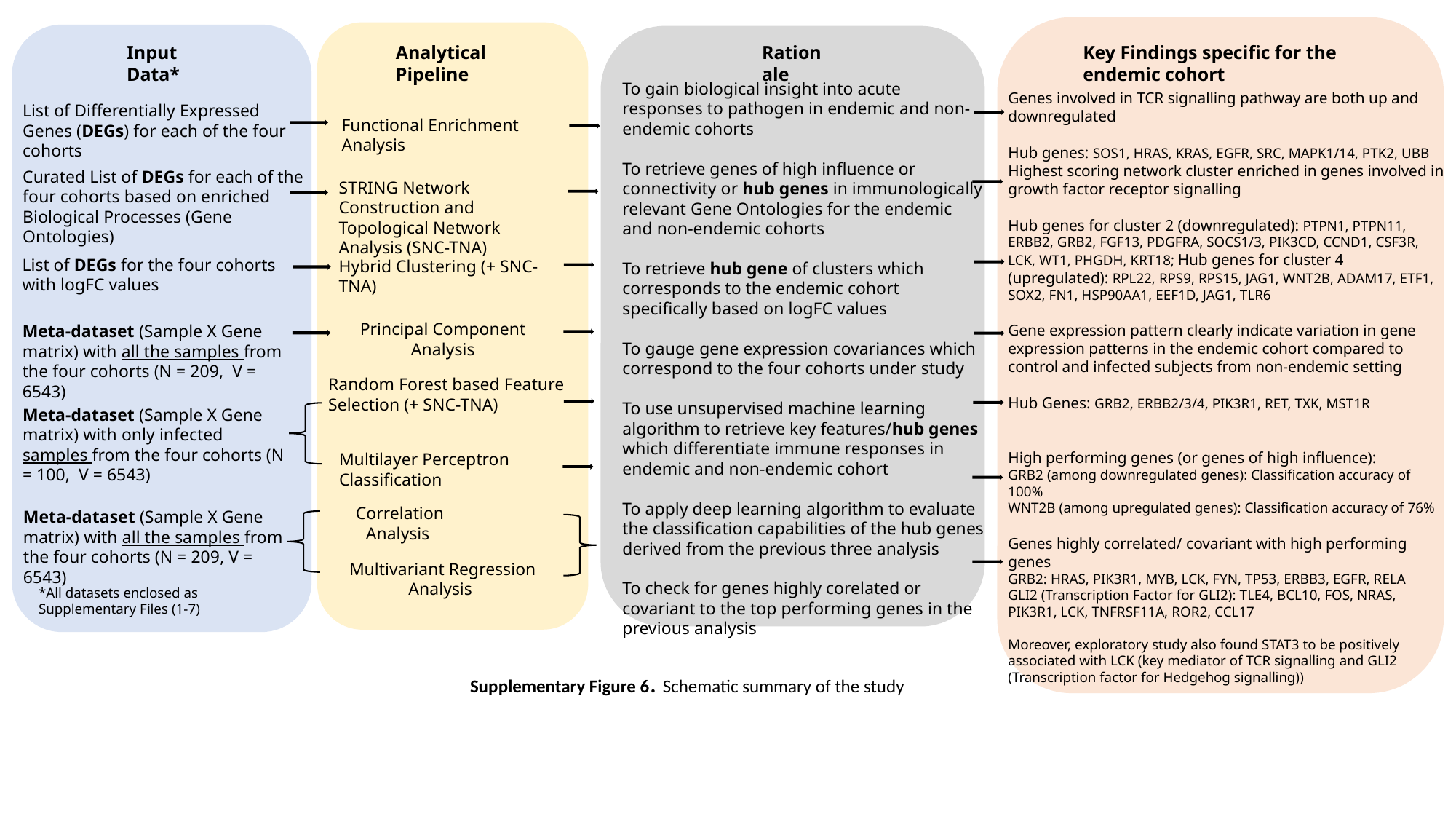

Key Findings specific for the endemic cohort
Input Data*
Analytical Pipeline
Rationale
To gain biological insight into acute responses to pathogen in endemic and non-endemic cohorts
To retrieve genes of high influence or connectivity or hub genes in immunologically relevant Gene Ontologies for the endemic and non-endemic cohorts
To retrieve hub gene of clusters which corresponds to the endemic cohort specifically based on logFC values
To gauge gene expression covariances which correspond to the four cohorts under study
To use unsupervised machine learning algorithm to retrieve key features/hub genes which differentiate immune responses in endemic and non-endemic cohort
To apply deep learning algorithm to evaluate the classification capabilities of the hub genes derived from the previous three analysis
To check for genes highly corelated or covariant to the top performing genes in the previous analysis
Genes involved in TCR signalling pathway are both up and downregulated
Hub genes: SOS1, HRAS, KRAS, EGFR, SRC, MAPK1/14, PTK2, UBB
Highest scoring network cluster enriched in genes involved in growth factor receptor signalling
Hub genes for cluster 2 (downregulated): PTPN1, PTPN11, ERBB2, GRB2, FGF13, PDGFRA, SOCS1/3, PIK3CD, CCND1, CSF3R, LCK, WT1, PHGDH, KRT18; Hub genes for cluster 4 (upregulated): RPL22, RPS9, RPS15, JAG1, WNT2B, ADAM17, ETF1, SOX2, FN1, HSP90AA1, EEF1D, JAG1, TLR6
Gene expression pattern clearly indicate variation in gene expression patterns in the endemic cohort compared to control and infected subjects from non-endemic setting
Hub Genes: GRB2, ERBB2/3/4, PIK3R1, RET, TXK, MST1R
High performing genes (or genes of high influence):
GRB2 (among downregulated genes): Classification accuracy of 100%
WNT2B (among upregulated genes): Classification accuracy of 76%
Genes highly correlated/ covariant with high performing genes
GRB2: HRAS, PIK3R1, MYB, LCK, FYN, TP53, ERBB3, EGFR, RELA
GLI2 (Transcription Factor for GLI2): TLE4, BCL10, FOS, NRAS, PIK3R1, LCK, TNFRSF11A, ROR2, CCL17
Moreover, exploratory study also found STAT3 to be positively associated with LCK (key mediator of TCR signalling and GLI2 (Transcription factor for Hedgehog signalling))
List of Differentially Expressed Genes (DEGs) for each of the four cohorts
Functional Enrichment Analysis
Curated List of DEGs for each of the four cohorts based on enriched Biological Processes (Gene Ontologies)
STRING Network Construction and Topological Network Analysis (SNC-TNA)
List of DEGs for the four cohorts with logFC values
Hybrid Clustering (+ SNC-TNA)
Principal Component Analysis
Meta-dataset (Sample X Gene matrix) with all the samples from the four cohorts (N = 209, V = 6543)
Random Forest based Feature Selection (+ SNC-TNA)
Meta-dataset (Sample X Gene matrix) with only infected samples from the four cohorts (N = 100, V = 6543)
Multilayer Perceptron Classification
Correlation Analysis
Meta-dataset (Sample X Gene matrix) with all the samples from the four cohorts (N = 209, V = 6543)
Multivariant Regression Analysis
*All datasets enclosed as Supplementary Files (1-7)
Supplementary Figure 6. Schematic summary of the study

## Slide 7
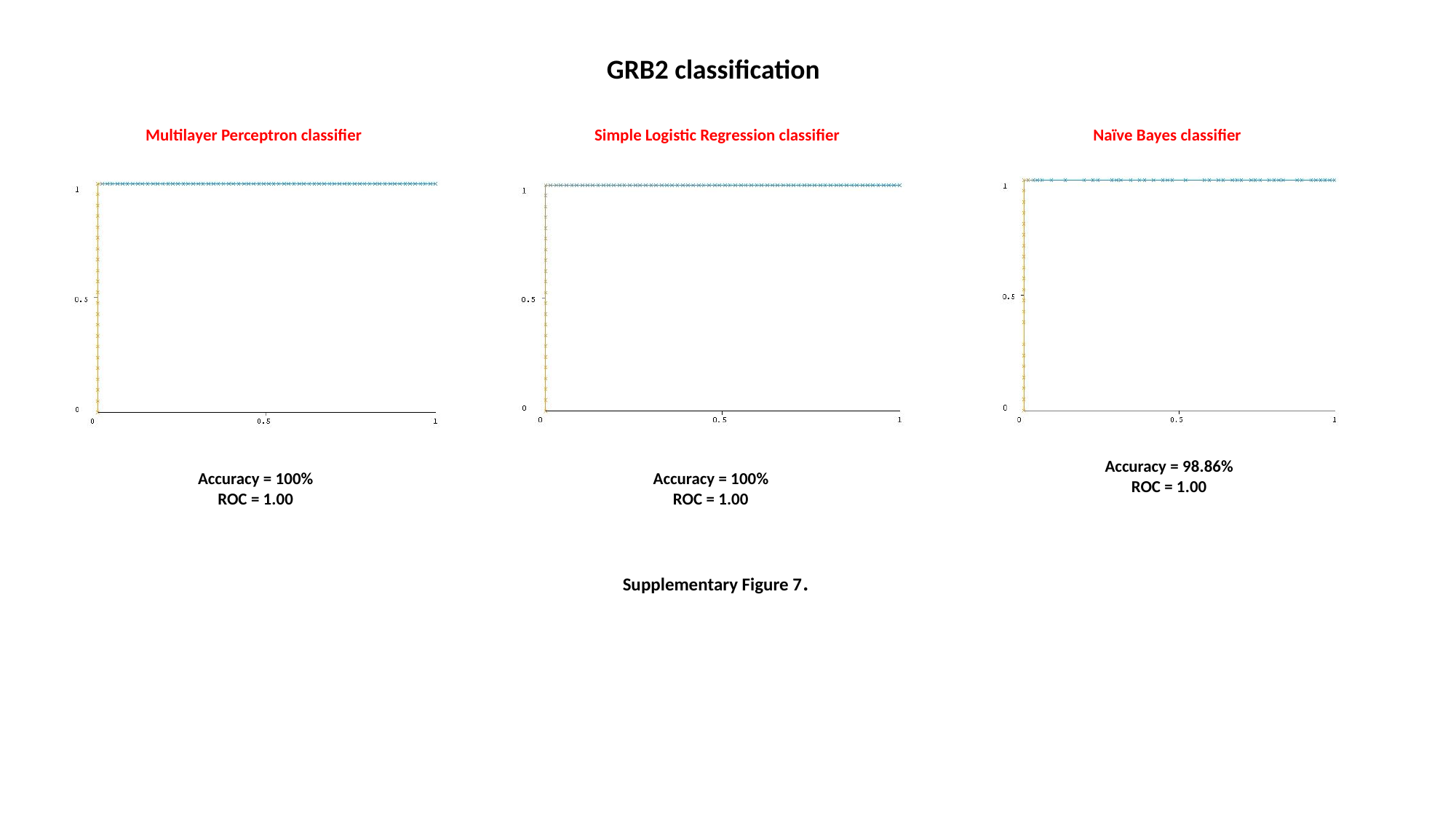

GRB2 classification
Multilayer Perceptron classifier
Simple Logistic Regression classifier
Naïve Bayes classifier
Accuracy = 98.86%
ROC = 1.00
Accuracy = 100%
ROC = 1.00
Accuracy = 100%
ROC = 1.00
Supplementary Figure 7.

## Slide 8
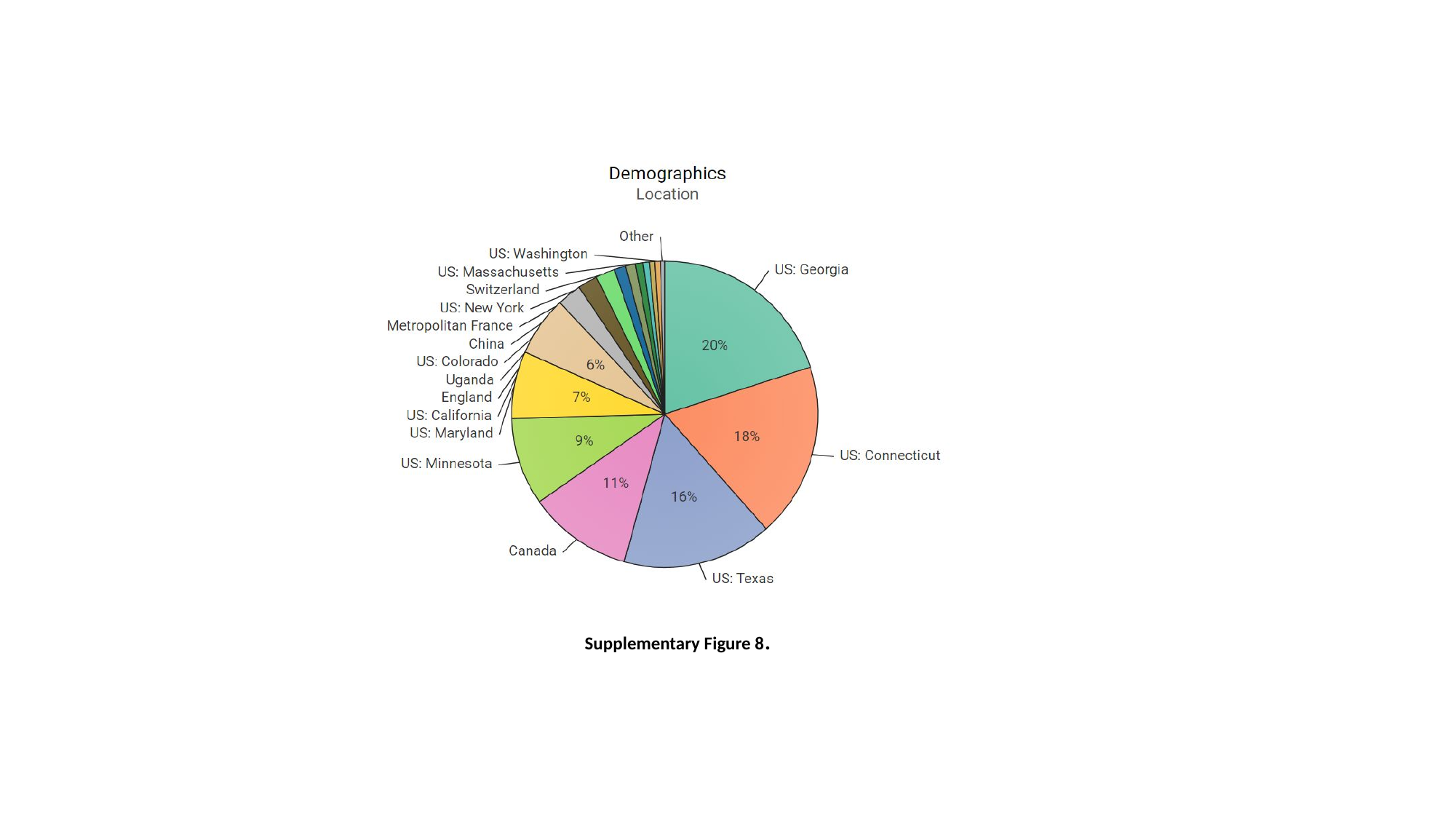

Supplementary Figure 8.
