# Supplementary material for: Systems and computational analysis of gene expression datasets reveals GRB-2 suppression as an acute immunomodulatory response against enteric infections in endemic settings
Source: Front Immunol. 2024 Feb 16;15:1285785. doi: 10.3389/fimmu.2024.1285785 (PMC10906661; doi:10.3389/fimmu.2024.1285785)
Supplement: Supplementary file 9 [file Table_7.docx]

**Table S1(a). Top 10 upregulated genes at the acute stage of each of the infection with their corresponding logFC values**

|  | **S. typhi (Vietnam)** | | **S. typhi (Oxford**) | | **ETEC** | | **Rotavirus** | |
| --- | --- | --- | --- | --- | --- | --- | --- | --- |
|  | Genes | logFC | Genes | logFC | Genes | logFC | Genes | logFC |
|  | CD22 | 6.37 | ANKRD22 | 5.23 | CD177 | 1.65 | G0S2 | 6.83 |
|  | AVPR1A | 5.95 | IDO1 | 4.22 | ARG1 | 1.38 | CXCL8 | 5.01 |
|  | CASP1 | 5.86 | SERPING1 | 4.12 | CEACAM1 | 1.35 | MAFF | 4.79 |
|  | S100A2 | 5.46 | FCGR1A | 3.75 | FCAR | 1.26 | CXCL2 | 4.21 |
|  | MTCH2 | 4.69 | SEPT4 | 3.62 | BMX | 1.24 | IFI27 | 4.15 |
|  | DNAJA3 | 3.9 | CXCL10 | 3.47 | FPR2 | 1.20 | RGS1 | 4.02 |
|  | ECHS1 | 3.83 | FCGR1B | 3.46 | PLSCR1 | 1.16 | IL6 | 3.98 |
|  | IFI6 | 3.77 | C1QB | 3.40 | ORM1 | 1.13 | CD83 | 3.8 |
|  | ATF6 | 3.64 | RSAD2 | 3.37 | IL1R2 | 1.01 | IL1B | 3.7 |
|  | RBPJ | 3.6 | IFI44L | 3.27 | CPD | 1.006 | DUSP2 | 3.14 |

**Table S1(b). Top 10 downregulated genes at the acute stage of each of the infection with their corresponding logFC values**

|  | **S. typhi (Vietnam)** | | **S. typhi (Oxford**) | | **ETEC** | | **Rotavirus** | |
| --- | --- | --- | --- | --- | --- | --- | --- | --- |
|  | Genes | logFC | Genes | logFC | Genes | logFC | Genes | logFC |
|  | CLUL1 | -5.29 | OLIG1 | -1.56 | CLC | -1.42 | HBB | -4.86 |
|  | HAAO | -5.16 | PIK3IP1 | -1.27 | PRKACB | -1.37 | HBA2 | -4.79 |
|  | TK1 | -4.57 | FCER1A | -1.46 | NMT2 | -1.13 | PPBP | -3.24 |
|  | LPAR1 | -4.52 | CACNA2D3 | -1.45 | KLF12 | -1.23 | SEPT5 | -3.1 |
|  | CXXC1 | -4.14 | TMEM8B | -1.26 | CD52 | -1.24 | ITGA2B | -3.02 |
|  | NHLH1 | -4.13 | DPEP3 | -1.34 | PAX5 | -1.22 | F13A1 | -2.96 |
|  | CLEC4E | -3.96 | PASK | -1.29 | RASGRP1 | -1.17 | CCR2 | -2.57 |
|  | NUP50 | -3.87 | FCGBP | -1.52 | OSBPL10 | -1.23 | NRGN | -2.5 |
|  | CCL27 | -3.71 | CLC | -1.38 | BCL11B | -1.19 | HBG1 | -2.41 |
|  | ATP11A | -3.7 | LRRN3 | -1.41 | RPL9 | -1.142 | IL16 | -2.26 |

**Table S2. KEGG intracellular signalling pathways significantly enriched at early time points each of the infections (FDR< 0.05)**

| **Intracellular signalling pathways** | **P-Value** | **Count** |
| --- | --- | --- |
| hsa04022: cGMP-PKG signalling pathway | 8.31418E-05 | 45 |
| hsa04020: Calcium signalling pathway | 9.82445E-05 | 59 |
| hsa04024: cAMP signalling pathway | 0.00023801 | 54 |
| hsa04010: MAPK signalling pathway | 0.000338657 | 67 |
| hsa04921: Oxytocin signalling pathway | 0.000493554 | 40 |
| hsa04919: Thyroid hormone signalling pathway | 0.000693116 | 33 |
| hsa04151: PI3K-Akt signalling pathway | 0.002136323 | 74 |
| hsa04072: Phospholipase D signalling pathway | 0.003299214 | 36 |
| hsa04014: Ras signalling pathway | 0.00359587 | 52 |
| hsa04630: JAK-STAT signalling pathway | 0.004108819 | 39 |
| hsa04071: Sphingolipid signalling pathway | 0.008635076 | 29 |
| hsa04015: Rap1 signalling pathway | 0.011423128 | 45 |
| hsa04621: NOD-like receptor signalling pathway | 1.75896E-09 | 19 |
| hsa04623: Cytosolic DNA-sensing pathway | 0.000677493 | 7 |
| hsa04612: Antigen processing and presentation | 0.001533792 | 9 |
| **Rotavirus (Non-endemic)** |  |  |
| hsa04064:NF-kappa B signalling pathway | 1.23503E-10 | 22 |
| hsa04657:IL-17 signalling pathway | 9.51909E-10 | 20 |
| hsa04010: MAPK signalling pathway | 3.3407E-06 | 29 |
| hsa04668: TNF signalling pathway | 2.96E-06 | 17 |
| hsa04621: NOD-like receptor signalling pathway | 3.62108E-06 | 22 |
| hsa04662: B cell receptor signalling pathway | 0.00019025 | 12 |
| hsa04620: Toll-like receptor signalling pathway | 0.004814287 | 11 |
| hsa04625:C-type lectin receptor signalling pathway | 0.004814287 | 11 |
| hsa04151: PI3K-Akt signalling pathway | 0.063743878 | 20 |
| hsa04612: Antigen processing and presentation | 0.024252176 | 8 |
| hsa04630: JAK-STAT signalling pathway | 0.085624208 | 11 |
| hsa04660: T cell receptor signalling pathway | 0.088318538 | 8 |

**Table S3. Hub genes derived from topological network analysis for each of the constructed network**

| **PPI Network** | **MCC** | | **DMNC** | | **BN** | |
| --- | --- | --- | --- | --- | --- | --- |
|  | **Node** | **Score** | **Node** | **Score** | **Node** | **Score** |
| **S. typhi (Vietnam)**  **Nodes:** 375  **Edges:** 1806 | SOS1 | 1.71E+10 | PDGFA | 0.818 | RELA | 34 |
|  | HRAS | 1.71E+10 | MAPK6 | 0.816 | SRC | 30 |
|  | KRAS | 1.71E+10 | ERBB3 | 0.804 | CTNNB1 | 30 |
|  | SRC | 1.71E+10 | FLT1 | 0.787 | HRAS | 22 |
|  | EGFR | 1.71E+10 | EPHB1 | 0.765 | AKT1 | 21 |
| **S. typhi (Oxford)**  **Nodes:** 84  **Edges:** 967 | IFIT5 | 2.75E+43 | IFIT5 | 1.457 | STAT1 | 12 |
|  | IFIT1 | 2.75E+43 | IFITM2 | 1.457 | DDX58 | 10 |
|  | ADAR | 2.75E+43 | ADAR | 1.457 | OAS2 | 9 |
|  | RSASEL | 2.75E+43 | RNASEL2 | 1.457 | CASP1 | 8 |
|  | BST2 | 2.75E+43 | BST2 | 1.457 | NOD2 | 8 |
| **Rotavirus**  **Nodes:** 171  **Edges:** 951 | TNF | 4.76E+07 | CCL20 | 0.881 | STAT1 | 15 |
|  | IL1B | 4.76E+07 | CCL3 | 0.821 | SRC | 13 |
|  | IL6 | 4.76E+07 | IFI27 | 0.811 | CD4 | 12 |
|  | CCL2 | 4.76E+07 | PSMB8 | 0.811 | EGFR | 11 |
|  | IL1A | 4.76E+07 | VCL | 0.758 | RELA | 11 |
| **ETEC**  **Nodes:** 38  **Edges:** 169 | PIK3CA | 61682 | CD79A | 0.763 | SHC1 | 12 |
|  | LCK | 55260 | CD79B | 0.763 | PIK3CA | 7 |
|  | CD28 | 53101 | CD2 | 0.758 | ITGB1 | 6 |
|  | CD247 | 51360 | CD247 | 0.754 | ITGB3 | 5 |
|  | CD4 | 47004 | LCK | 0.744 | PLAUR | 4 |

**Table S4. Top 5 network clusters obtained from the four extended PPI network along with key regulators/transcription factors for the cluster derived from TRRUST database**

| **Cohort**  **(Score)** | **Genes** | **Enriched Pathways**  **(strength > 2, gene count >5, Top 5)** | **Transcription Factors** |
| --- | --- | --- | --- |
| **S. typhi (Oxford) cohort** (40.55) | IRF1, IRF2, IRF3, IRF4, IRF5, IRF7, IRF9, IFIT1, IFIT2, IFIT3, IFIT5, IFI6, IFI27, IFI35, IFITM1, IFITM2, IFITM3, STAT1, STAT2, HLA-A, HLA-B, HLA-C, HLA-E, HLA-F, HLA-G, OAS1, OAS2, OAS3, OASL, RNASEL, MX1, MX2, RSAD2, BST2, ISG15, ISG20, GBP2, SAMHD1, XAF1, ADAR, PSMB8 | Interferon alpha/beta signalling (**HSA-909733**), Endosomal/ Vacuolar pathway **(HSA-1236977**), OAS antiviral response (**HSA-8983711**), Antigen Presentation: Folding, assembly and peptide loading of class I MHC(**HSA-983170**), Interferon gamma signalling (**HSA-877300**) | HIVEP2, **CIIT2**, MYCN, **JUN**, **STAT1**, STAT2, STAT3, STAT6, MYC, **BRCA1**, **IRF1**, IRF3, **RELA**, CREB5, **PITX1**, NFKB1, **SP1** |
| **S. typhi (Vietnam)** **cohort** (18.2) | PIK3CA, PIK3R1, PIK3CB, EGF, EFGR, ERBB2, ERBB3, PTPN11, LCK, FYN, MAPK1, MAPK3, RHOA, GRB2, PTK2, SOS1, SHC1, HRAS, KRAS, JAK2 | Signalling by FGFR3 fusions in cancer (**HSA-8853334**), Signalling by PDGFRA transmembrane, juxta-membrane and kinase domain mutants (**HSA-9673767**), Activated NTRK2 signals through RAS (**HSA-9026519**), Signalling by FGFR4 in disease (**HSA-5655291**), Constitutive Signalling by Overexpressed ERBB2 (**HSA-9634285**) | **MYB, SP1** |
| **Rotavirus** **cohort** (13.385) | IL1A, IL1B, IL6, TNF, IL10, CCL1, CCL2, CCL3, CCL4, CCL5, CCL20, CXCL1, CXCL2, CXCL8, CXCL10 | Interleukin-10 signalling **(HSA-6783783**), Chemokine receptors bind chemokines (**HSA-380108**) | **RELA**, NFKB1, REL, **JUN**, **STAT1**, CEBPB, SPI1, **IRF3**, CEBPD, NFKBIA, XBP1, **SP1**, ATF, STAT3, VDR, HDAC11, ZNF300, CEBPA, IRF1, ZFP36, IRF8, IRF7, EGR1, CREB1, **HMGA1**, DDIT3, AHR, E3F1, PARP1, NR1I2, HDAC2, HSF1, JUND, GATA3, KLF, SIRT1, EP300, FOS |
| **ETEC cohort** (8.33) | CD4, CD2, CD28, CD8A, CD19, CD79A/B, CD86, CD80, PIK3R1, PIK3CA, LCK, PRKCQ | CD28 dependent PI3K/Akt signalling (**HSA-389357**) | TRERF1, MYB, **RELA**, NFKB1, ETS1 |
| **Rotavirus cohort** (9.241) | IFI6, IFI27, OAS1, OAS2, OASL, IFIT3, STAT3, ISG15, JAK2, PIK3R1, PIK3CA, PTK2, PLCG1, NFKBIA, MAPK3, MAPK14, VLC, RAC1, CXCR4, CREBBP, HDAC1, JUN, EGR1, VEGA, HIF1A, PSMB8, HCK, BLNK, SRC, EP300, PSMB8 | - | **BRCA1**, **RELA**, HIF1A, YY1, NFKB1, ZNF382, SPI2, MEF2A, KLF5, **STAT1,** PML, ARNT, VHL, MYC, FLI1, FOXO1, HDAC3, DNMT1, **STAT3**, USF2, HDAC, ESR1, ETS1, EGR1, CREB1, AR, TP53, **SP1** |

**Table S5. Genes/Features derived from different algorithms**

| **Feature Selection Source** | **Genes** |
| --- | --- |
| **Hybrid Clustering (Cluster 2)** | ABCB11, ADD1, ANPEP, ANXA1, AP2A2, AQP3, ARG2, ASNS, ATP6AP2, ATP6V1G2, BBS9, BCLAF1, BUB3, CACNA1G, CCND1, CD44, CDK16, CDK2, CELF2, CFDP1, CFTR, CHST1, CIAO1, CSF3R, CST6, CTSB, DHX34, DIMT1, DRAP1, E2F4, EDN3, EIF3E, EPM2A, ERBB2, ESR2, EXPH5, FAM20B, FARP2, FGF12, FGF13, FOXH1, FRY, GDF5, GM2A, GPM6B, GPR176, GRB2, GRIK3, GRSF1, GSTM5, HBP1, HERC4, HNRNPC, KLF1, KLHDC10, KLK2, KNG1, KRT18, LAMA2, LAMC2, LCK, LDLR, LGMN, LMNB1, LPAR1, MAPRE2, MECOM, MPZL2, MYL4, N4BP1, NACA, NCOA2, NEMP1, NFYB, NR3C1, NRP2, NUMA1, OR7E24, PAICS, PDGFRA, PDHB, PDK3, PHGDH, PIK3CD, PIP4K2A, PITX2, PKNOX1, PLCB1, PNN, POU2F2, PPFIA1, PPP1CA, PPP2R1A, PPP3R1, PRB1, PREPL, PRICKLE3, PRMT1, PTPN1, PTPN11, PTTG1, RAB11A, RAB11FIP3, RAB33A, RBMXL2, RECK, RGS6, RND2, RPLP1, RPS6KA3, RRP7A, RTN1, SACS, SCARB1, SERPINA1, SETD1A, SLC24A1, SLC29A1, SMG7, SOCS1, SOCS3, SPAG5, SPCS2, TBX5, TLK2, TLN1, TLR1, TLR2, TNFRSF21, TNK2, TRAF2, TRAF3IP2, TRIM37, UBE2D2, USP10, UTF1, VCL, VPS13A, WT1, WWP2, YKT6, ZC3H13, ZNF324, ZNF460, ZNF500 |
| **Hybrid Clustering (Cluster 4)** | ADAM17, ADD3, AKAP12, AKAP13, APLP2, ARHGAP1, ARHGEF12, ARIH1, ATP11B, ATP2A3, ATP4A, ATP6V0E1, ATXN2, BAG5, BAX, BCL6, BID, BTN3A2, CA2, CACNA1C, CACNB2, CALB1, CALD1, CAMSAP2, CBX6, CCR4, CD40LG, CDKN2B, CELSR2, CELSR3, CNBP, CR2, CUX1, CXADR, CYP4F2, DCT, DIAPH1, DKK3, DMPK, DNAJC16, DOCK3, DTNA, EDA, EEA1, EEF1D, ERCC1, ERCC4, ETF1, F2RL2, F7, FAM168A, FARP1, FBLN1, FCER1A, FGFR3, FN1, FSHB, GBE1, GCDH, GFRA1, GJA1, GLI1, GLUD1, GPC5, GPR19, GRB10, GTF3C1, HLA-DPB1, HLA-DRB6, HNRNPUL1, HOXA10, HOXA11, HSP90AA1, IL10RB, IRAK3, JAG1, KAT6B, KHK, KLK7, KRT1, LDOC1, LLGL2, MAP2, MAP2K6, MAPK11, MAZ, MDN1, METAP2, MID1, NDC80, NFRKB, NOP14, NRG1, NRGN, NTN3, NTRK2, OFD1, OPTN, PARVB, PDCD10, PDE4B, PDE6C, PDGFA, PGM3, PGR, PHYH, PHYHIP, PLIN3, PPP2CB, PPP2R5B, PPP3CC, PPP6C, PRDX6, PRKDC, PRKG2, PRMT2, PSIP1, PSMA3, PSMB2, PTPRC, RAB31, RABGGTA, RAE1, RASGRP2, RBBP4, RBM12, RBPMS, RCBTB2, RER1, RGS13, RNF103, RNF40, RNMT, RPL22, RPL6, RPS15, RPS16, RPS9, S100A2, SACM1L, SAT1, SCN2A, SERPINA7, SF3B1, SHH, SLC25A12, SLC25A3, SLC35A2, SLK, SMARCB1, SNAI2, SOX2, SPAM1, STAT1, SYNGR2, TACSTD2, TAPBP, TFE3, TGM3, THRA, TLE4, TLR6, TNC, TNFAIP8, TP53I11, TRO, TUBB3, USP19, USP4, WFS1, WNT2B, YAF2, YIPF3 |
| **Random Forest Algorithm** | AATF, ABL2, AIRE, ALOX15B, ANXA11, AP3D1, APBB2, APOB, ARF6, ARG2, ASCL2, ATF7, BATF, BAX, BAZ1B, BCL10, BCL2L11, BTC, C1S, CAMTA1, CASP1, CCL27, CD22, CD40, CD74, CD80, CEBPD, CELSR1, CFTR, CHRD, CHRNB2, CHST1, CNTF, COPS8, CPNE1, CPNE3, CR2, CREG1, CRISP3, CRLF1, CRX, CSNK1A1, CSNK1G2, CSPG4, DDN, DNAJA3, DPEP1, E2F4, EIF2B5, EIF5A, EPHA2, EPO, ERBB2, ERBB3, ERBB4, ETV4, F2, F2RL3, FDX1, FGF12, FGFR2, FGR, FLT4, FOXK2, GALR2, GCNT1, GCNT2, GRB2, GRIN1, HGF, HTR1D, IER2, IFNB1, IGLL1, IL1RN, ING1, INHA, INHBB, IRF1, KSR1, LAMC2, LGALS9, LPAR1, LRP4, LTB, MADCAM1, MAP2K4, MAP4K1, MST1R, MSX2, N4BP1, NFKB2, NHLH1, NKX2-1, NONO, NR1I3, NR4A3, NTRK2, OR2H2, P2RX4, PAK3, PGLYRP1, PIK3C2A, PIK3CB, PIK3R1, PKNOX1, PLCB1, POU2F2, POU4F2, PPIB, PPP2R1A, PRKACA, PRKCD, PSEN1, PSMA3, PTH1R, PTPN1, PTPRD, RASGRP3, RBPJ, RET, RPS6KA4, RRAGA, S100A9, SASH1, SERPINE1, SP2, TBKBP1, TBX1, TEAD1, TEAD4, TFAP2B, TFAP2C, TFDP2, TFR2, THRB, TIMP2, TMSB4X, TNFAIP6, TRAF2, TRIM31, TSPAN6, TXK |
| **Random Forest with PCA** | AATF, ALOX15B, ANXA11, APOB, ARF6, ARG2, BATF, BAX, BAZ1B, BCL10BCL2L11, CASP1, CCL27, CD22, CFTR, CHST1, CNTF, CR2, CRLF1, CSNK1A1, CSNK1G2, CSPG4, DNAJA3, E2F4, EIF2B5, EIF5A, EPHA2, ERBB3, ERBB4, F2RL3, FDX1, FGR, FLT4, GRB2, IL1RN, LPAR1, LRP4, MST1R, NHLH1, NTRK2, P2RX4, PIK3C2A, PIK3CB, PIK3R1, PKNOX1, PLCB1, PPP2R1A, PSMA3, PTPN1, RBPJ, RRAGA, SASH1, TEAD1, TFAP2B, TFDP2, THRB, TMSB4X, TNFAIP6, TRIM3 |

**Table S6. Curated GO terms from each of the modules derived from the common correlation matrix from Pipeline 2 of the analysis**

| **Module** | **GO Terms** | **P value** | **Immune response Genes** | **Regulators** |
| --- | --- | --- | --- | --- |
| **Module 3:** WNT and TCR signalling pathway | GO:0038095~Fc-epsilon receptor signalling pathway | 0.0012 | PSMA3, PSMB5, PLCG1, MAP2K7, MS4A2, PSMB10 | GTF2A1, ATF2, UCN, LDB1, MAML1, TNKS, PTH, HNF4G, SUPT4H1, CRX, ARID4A, WASL, FOXO3, RPS6KA5, ABLIM3, ZNF507, HMGN3, PELP1, EIF5A, KDM6B, SREBF1, NPM1, PROP1, RIPK2, NR1H2, NR2F1, NFATC1, SOX12, KLF4, ZFY, NFATC4, POMC, KLF6, TNIP1, MAFF, TCEA2, PRKD2, ITGB1BP1, SSBP2, PFKM, PSMD10, KHDRBS1, ATF2, LDB1, SHOX2, ZBTB1, SUPT4H1, ARID4A, FASLG, FOXO3, RNF2, CUX2, MTA1, EDNRB, RBBP4, HESX1, RBM10, ZNF266, TLE4, SREBF1, PROP1, ZNF282, NR1H2, PCGF2, USP2, NR2F1, SOX12, KLF4, NFATC4, CDK6, MAFF, SREBF1, ATF2, NR1H2, ZBTB1, TAF4, GTF2A1, TSC22D1, ABLIM3, LDB1, PTH, ARID4A, TRIP11, KLF4, TAF4, NFATC4 |
|  | GO:0070374~positive regulation of ERK1 and ERK2 cascade | 0.0015 | NTRK1, SEMA7A, RAMP3, NPY5R, RIPK2, NPY, GCNT2, NPTN, PRKD2, MAP2K7, CCL17, SSTR4 |  |
|  | GO:0051092~positive regulation of NF-kappaB transcription factor activity | 0.0019 | NTRK1, PRKCI, NPM1, RPS6KA5, RIPK2, TRIM25, S100A12, PRKD2, BCL10, TNFRSF11A |  |
|  | GO:0060071~Wnt signaling pathway, planar cell polarity pathway | 0.0024 | PSMA3, PSMB5, FZD7, ROR2, PSMB10, CELSR3 |  |
|  | GO:0043507~positive regulation of JUN kinase activity | 0.0057 | PTPN1, MAP4K2, TNFRSF11A, ROR2, MAP2K7 |  |
|  | GO:0060070~canonical Wnt signalling pathway | 0.0075 | EDNRB, WNT2B, PROP1, FZD7, SIAH2, HESX1, KLF4 |  |
|  | GO:0007254~JNK cascade | 0.0182 | ATF2, MAP4K2, RIPK2, ROR2, MAP2K7 |  |
|  | GO:0090090~negative regulation of canonical Wnt signaling pathway | 0.0242 | TLE4, PSMA3, PSMB5, SIAH2, ROR2, FOXO3, PFDN5, PSMB10 |  |
|  | GO:0043950~positive regulation of cAMP-mediated signalling | 0.0360 | POMC, UCN, GPR3 |  |
|  | GO:0070498~interleukin-1-mediated signalling pathway | 0.0396 | PSMA3, RPS6KA5, PSMB5, PSMB10 |  |
|  | GO:0000165~MAPK cascade | 0.0495 | PSMA3, NRAS, PSMB5, MBP, MAP2K7, PSMB10, MAPK4 |  |

**Supplementary Table 7.** Studies which report GRB2 downregulation in the ImmuneSpace database

| **Study Accession ID** | **Study Site** | **Age** | **Vaccine Administered** | **Time-point** | **Mean fold change** | **PubMed ID** |
| --- | --- | --- | --- | --- | --- | --- |
| SDY299 | US | 18-70 | HEPLISAV (against Hepatitis) | Day 1 | -1.99 | [22342916](http://www.ncbi.nlm.nih.gov/entrez/query.fcgi?cmd=Retrieve&db=pubmed&dopt=Abstract&list_uids=22342916), [23727002](http://www.ncbi.nlm.nih.gov/entrez/query.fcgi?cmd=Retrieve&db=pubmed&dopt=Abstract&list_uids=23727002),  [26067185](http://www.ncbi.nlm.nih.gov/entrez/query.fcgi?cmd=Retrieve&db=pubmed&dopt=Abstract&list_uids=26067185),  [29289383](http://www.ncbi.nlm.nih.gov/entrez/query.fcgi?cmd=Retrieve&db=pubmed&dopt=Abstract&list_uids=29289383) |
| SDY1328 | Canada | 25-40 or >65 | Twinrix (Hepatitis A/B), Td booster (Tetanus/Diphtheria) and Dukoral (Cholera) | Day 7 | -1.56 | [26742691](http://www.ncbi.nlm.nih.gov/entrez/query.fcgi?cmd=Retrieve&db=pubmed&dopt=Abstract&list_uids=26742691) |
| SDY1276 | US | 18-40 | Trivalent influenza vaccine | Day 1 | -0.76 | [23878721](http://www.ncbi.nlm.nih.gov/entrez/query.fcgi?cmd=Retrieve&db=pubmed&dopt=Abstract&list_uids=23878721)  [21357945](http://www.ncbi.nlm.nih.gov/entrez/query.fcgi?cmd=Retrieve&db=pubmed&dopt=Abstract&list_uids=21357945) |
| SDY180 | US | 18-65 | Pneunomax23 | Day 6 | -0.60 |  |
